# Dry mouth in palliative care: A systematic review of clinical practice guidelines around the world

**DOI:** 10.1177/02692163261434188

**Published:** 2026-04-29

**Authors:** A. I. van der Meulen, A. Stoppelenburg, M. Theunissen, E. J. M. de Nijs, M. H. J. van den Beuken-van Everdingen, Y. M. van der Linden

**Affiliations:** 1 Centre of Expertise in Palliative Care, Leiden University Medical Centre, Netherlands; 2 Centre of Expertise in Palliative Care, Maastricht University Medical Centre+, Netherlands

**Keywords:** xerostomia, palliative care, frailty, practice guideline

## Abstract

**Background:**

Dry mouth is a disruptive symptom in patients with life-limiting illnesses. It has one of the highest symptom burdens in palliative care and a significant impact on quality of life. Nonetheless, dry mouth remains an underacknowledged and undertreated symptom with limited evidence-based interventions.

**Aim:**

To examine the quality and content of guidelines for the treatment of dry mouth in patients with life-limiting illnesses.

**Design:**

A systematic review of clinical practice guidelines was conducted (preregistered in PROSPERO in 2023). PRISMA reporting guidelines were followed. The search strategy involved 4 scientific databases and 9 guideline databases, targeted searches and stakeholder outreach for 183 countries/regions. Quality and content were analysed using the AGREE II instrument and directed content analysis, respectively.

**Data sources:**

Seventy-two clinical practice guidelines from 42 countries across 6 continents were included.

**Results:**

Only two guidelines were recommended for use based on the AGREE II quality appraisal. Four main themes emerged from the content analysis: (1) Assessment of dry mouth by medical history, oral examination and measuring instruments (*n* = 32, *n* = 36 and *n* = 19 guidelines respectively); (2) Oral care (*n* = 68); (3) Management by treating causes (*n* = 40), saliva substitutes (*n* = 66) and stimulants (*n* = 62), diet (*n* = 45), and medication (*n* = 40); and (4) Patient and family involvement (*n* = 43).

**Conclusion:**

Despite differences in quality and comprehensiveness of guidelines for treatment of dry mouth in palliative care, many care practices are shared worldwide. This review highlights the need for methodologically robust guidelines with a strong evidence base that specifically focus on dry mouth.

**What is already known about the topic?**

- Dry mouth is a prevalent and severely distressing symptom in patients with life-limiting illnesses.
- Dry mouth is underacknowledged by patients, health care professionals, and in scientific research, leading to inadequate treatment.
- Clinical practice guidelines in palliative care are a key facilitator for improving both knowledge and care practices, including symptom management of dry mouth.

**What this paper adds?**

- Quality of guidelines for dry mouth is limited, and most recommendations are based on expert opinion rather than (graded) scientific evidence.
- Few guidelines fully focus on oral problems (including dry mouth) in palliative care, and most recommendations are non-specific in nature.
- While the methodological quality and comprehensiveness vary widely across guidelines, there is a high level of consistency among recommendations for the assessment of dry mouth, the role of oral care, the management of dry mouth, and the patient and family involvement in dry mouth care.

**Implications for theory, practice or policy**

- This study highlights the necessity of developing methodologically robust, easy-to-use guidelines that specifically focus on dry mouth in palliative care.
- Guidelines for dry mouth should include detailed, practical recommendations for the assessment and management of dry mouth, adapted to local context, and with an increased focus on the psychosocial dimension of dry mouth.
- Future research should focus on evaluating current dry mouth care practices in high quality clinical trials in the palliative care setting, and on identifying new dry mouth treatment options.

## Introduction

Dry mouth is a common and disruptive symptom in people in all phases of palliative care, and with different underlying life-limiting conditions, such as advanced cancer, severe systemic diseases, and geriatric frailty.^1^ Dry mouth may result from reduced salivary flow due to salivary gland hypofunction (hyposalivation) or occur as a subjective sensation of oral dryness (xerostomia) that is not always explained by decreased saliva production.^2^ It is present in 40%–83.5% of patients with advanced cancer,^3–6^ and up to 91% in terminally ill cancer patients.^7^ Similarly, 40%–70% of frail older people suffer from dry mouth,^8–10^ around 60% of patients with advanced COPD or heart failure,^11,12^ 28%–74% of patients with end-stage renal disease^13^ and 59% of hospitalised HIV/AIDS patients.^14^

Dry mouth is a highly disruptive symptom in daily life.^1,15,16^ It can have a severe functional impact on swallowing, eating, sleeping, and speaking, and may lead to pain, infection, halitosis and other oral symptoms.^15,17–20^ Dry mouth is also known to have a significant psychosocial impact and has one of the highest symptom burdens for patients with advanced diseases.^6,16,21^ It affects patients’ social interaction and intimacy,^17–19^ leads to feelings of shame and loneliness,^18,22^ and is associated with anxiety and depression.^21,22^

Dry mouth is an underacknowledged and undertreated symptom by patients, family caregivers, and healthcare professionals. Few patients report their dry mouth, perceiving their symptoms as an inevitable consequence of their disease or treatment, and being unaware of available treatment options.^19,23^ Healthcare professionals do not systematically or appropriately assess oral health and dry mouth in patients with life-limiting illnesses.^19,24–26^ They report a lack of knowledge and confidence in conducting oral health consultations and in providing oral care.^25,26^ Furthermore, healthcare professionals have reported to consider dry mouth to be someone else’s responsibility and a low priority condition.^23,26,27^ Suggested reasons for this include limited formal training and a lack of uniform guidelines.^23,24,26,27^ In addition, scientific evidence for the effect of dry mouth interventions is limited and of low quality, both in general^28–30^ and for patients with life-limiting illnesses.^31,32^ Therefore, an overview of current best practice is needed to provide a foundation for clinical practice and future research. Based on the best available evidence, best practices can be found in clinical practice guidelines.

Clinical practice guidelines are used to formalise and standardise clinical practice with the aim of improving quality of care.^33,34^ They assist health care professionals’ decision-making, as well as highlight areas of (in)effective treatment.^34,35^ While there is no evidence yet for dry mouth guidelines specifically, both the World Health Organisation and previous research have suggested that palliative care guidelines and oral health guidelines can improve awareness, knowledge and effectiveness of care practices.^33,36–39^

To our knowledge, no formal evaluation has been conducted to analyse the quality and content of clinical practice guidelines for dry mouth in palliative care. This review therefore aims to provide insight into current standards for care practices for dry mouth in patients with life-limiting illnesses.

Specifically, the aim of this review is to:

1. Analyse the quality of clinical practice guidelines for dry mouth in patients with life-limiting illnesses.
2. Analyse the content and consistency of clinical practice guideline recommendations for the assessment and treatment of the symptom dry mouth in patients with life-limiting illnesses.

## Methods

### Design

This systematic review was conducted based on the recommendations for reviews of clinical practice guidelines from Johnston et al. (2019)^40^ and reported following the Preferred Reporting Items for Systematic Reviews and Meta-Analyses (PRISMA) protocol.^41^ The review was pre-registered using the International Prospective Register of Systematic Reviews (PROSPERO, CRD42023415285).^42^

### Search strategy

Multiple search processes were employed to ensure a wide international coverage of clinical practice guidelines. The search strategy combined two search strings related to (1) dry mouth and (2) clinical practice guidelines. In addition, search strings related to (3) oral care were included in all search processes except the scientific database searches (see Supplemental File 1).

First, a systematic literature search in four scientific databases (PubMed, CINAHL, Cochrane Library and EMBASE) was conducted with an information specialist (first search November 2023, updated August 2025).

Second, nine guideline databases were searched (Trip Pro, BIGG International Database of GRADE guidelines, ECRI Guidelines Trust, GIN International Guidelines Library, Scottish Intercollegiate Guidelines Network (SIGN), U.S. Preventive Services Task Force (USPSTF) Database, Arbeitsgemeinschaft der Wissenschaftlichen Medizinischen Fachgesellschaften (AWMF) Leitlinien Register, WHO Guidelines, GRADEpro GDT database).

Third, targeted searches were performed for 183 countries and other territories across six continents (Europe, Asia, North America, South America, Africa, Oceania). These countries were selected based on the list of EAPC members, the directory of the International Association for Hospice and Palliative Care (IAHPC),^43^ the APCA Atlas of Palliative Care in Africa,^44^ the EAPC Atlas of Palliative Care in Europe,^45^ the Atlas of Palliative Care in Latin America^46^ and the Atlas of Palliative Care in the Eastern Mediterranean region.^47^ Searches were conducted in the language of origin for each country and in English.

Fourth, at least one key stakeholder per country (if available) was approached via email to request any clinical practice guidelines, and/or to confirm the use of a clinical practice guideline found in one of the other search steps. This stakeholder was preferably a national representative for palliative care, identified from the IAHPC directory,^43^ the Palliative Care Atlases^44–47^ or a key scientific publication (see Supplemental File 2 for more information on the targeted searches & stakeholder outreach).

Fifth, additional clinical practice guidelines were searched by screening the reference lists in the included guidelines.

### Eligibility criteria & screening

The PICAR framework (Population, Intervention(s), Comparator(s), Attributes of eligible clinical guidelines and Recommendation characteristics)^40^ was used to define the criteria for eligibility (Table 1).

**Table 1.** PICAR framework^40^ for systematic reviews.

Study-specific criteria	
**P**opulation & clinical indication	• Adult (> 18 years) patients with a life-limiting illness (all phases of palliative care) • Dry mouth and/or xerostomia
**I**ntervention(s)	• All recommendations and interventions for dry mouth are of interest, including but not limited to: ○ Diagnostics (anamnesis, examination); ○ Prevention; ○ Treatment; ○ Patient Education.
**C**omparator(s)	• No comparator.
**A**ttributes of eligible clinical practice guidelines	• **Year:** 2000–2024. • **Version:** latest freely available version. • **Scope:** primary focus on life-limiting illness. • **Rigour:** preferably evidence-based and systematically developed, but all types are included. • **Target population:** any health care professionals that work with adult patients with a life-limiting illness. • **Coverage:** continental, national or (larger) regional; not institutional. • **Language:** no restrictions. • **Recommendations:** ○ At least one recommendation for dry mouth. ○ Exclusion: when recommendation is explicitly stated to be for dry mouth due to Sjogren’s syndrome or radiotherapy-induced dry mouth.
**R**ecommendation characteristics	• No specific restrictions placed on levels of confidence (e.g. GRADE methodology), comparators or location of recommendation. • Recommendations must explicitly discuss ⩾1 concrete diagnostics, prevention, treatment or patient education action for dry mouth.

Five main criteria were used during screening:

1. Document is a clinical practice guideline. The frequently used clinical practice guideline definition by Lohr et al. 1999 was considered as a starting point: ‘*systematically developed statements to assist practitioner and patient decisions about appropriate health care for specific clinical circumstances’.*^48^ To allow for guidelines with varying levels of quality and comprehensiveness, our interpretation of a clinical practice guideline was expanded to include guidelines that may not have a systematic review underpinning the recommendations but have been developed and endorsed by a (national) professional organisation, or group of experts.
2. Document provides recommendation(s) for dry mouth. Clinical practice guidelines with recommendations explicitly targeted at dry mouth caused by Sjogren’s syndrome or radiotherapy for head and neck cancer were excluded. If guidelines also addressed dry mouth in broader populations, they were included, but any recommendations specific to radiotherapy-induced or Sjogren’s-related dry mouth were not extracted or coded (unless mentioned otherwise).
3. Document is related to adult patients (over 18 years) in palliative care (as defined by the WHO definition).^49^ This included patients in all phases of palliative care and with varying life-limiting illnesses, with no restrictions on type of disease, prognosis or life stage. Eligible guidelines encompassed palliative care, end-of-life and terminal care in general, but also advanced diseases (including but not limited to cancer) and geriatric frailty.
4. Document has a regional, national or international coverage. There were no restrictions based on language of the clinical practice guideline.
5. Document is published after the year of 2000, and is the latest version available and accessible.

Potential clinical practice guidelines were screened by two independent researchers (AM and AS), both with a background in palliative care (as a medical doctor and researcher, and as former nurse and post-doctoral researcher, respectively). Clinical practice guidelines were screened independently using Rayyan.ai. After screening, any disagreements were discussed until consensus was reached. When necessary, the wider research group was involved to reach consensus, which consisted of two palliative care specialists (radiation oncologist (YL) and internal medicine specialist (MB), and one post-doctoral palliative care researcher (MT)).

### Data extraction

All relevant supporting documents were retrieved before data extraction and analysis. Data extraction was completed by one researcher (AM) and verified by an independent reviewer for accuracy and completion (AS). General characteristics of each included clinical practice guideline were recorded, such as title, author information, year of publication, and target population.

### Quality appraisal

The Appraisal of Guidelines for Research and Evaluation Instrument version 2 (AGREE II, online portal: AGREE Plus) was used to assess the quality of the clinical practice guidelines.^35^

The 23-item AGREE II tool considers the quality of the clinical practice guideline in terms of scope and purpose, stakeholder involvement, rigour of development, clarity of presentation, applicability and editorial independence. Reviewers score each item from strongly disagree to strongly agree (seven-point Likert scale), after which scaled domain scores are calculated. In addition, a ‘global judgement’ about the clinical practice guideline is made by reviewers to assess the perceived overall quality and recommendation of the clinical practice guideline.^35^ Two reviewers (AM, AS) independently applied the AGREE II tool to all clinical practice guidelines. Rater reliability was measured by a two-way mixed effects intraclass correlation coefficient (ICC) for consistency of agreement, using IBM SPSS Statistics v25.

### Directed content analysis

To assess the consistency of recommendations in the clinical practice guidelines and identify overarching themes and potential content gaps a directed content analysis was used.^50^ After familiarisation with the included clinical practice guidelines, key components of all clinical practice guidelines were independently identified by AS and AM and used as the foundation of the coding framework. This initial deductive approach was then supplemented by inductively adding and adapting codes during the coding process. ATLAS.ti software (v25) was used to collect and analyse the content of the clinical practice guidelines. Clinical practice guidelines were analysed using the English translation of the original document.

## Results

**Figure 1.**
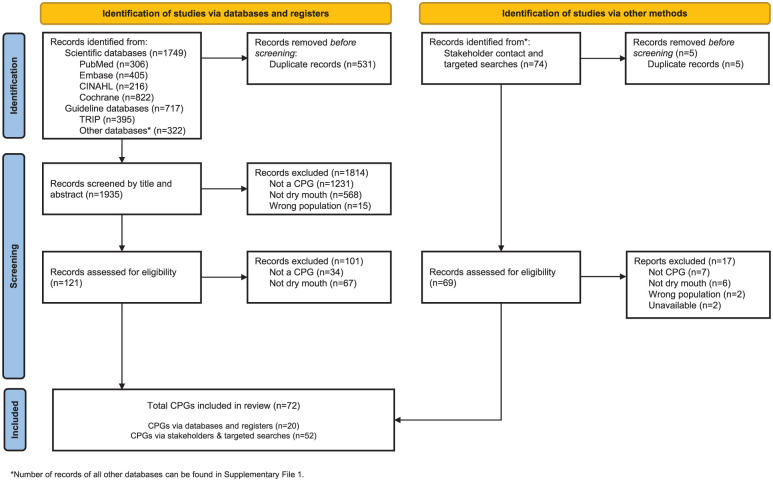
PRISMA flowchart of included clinical practice guidelines (*n* = 72).

Searches retrieved 1749 records from the scientific databases, 717 from the guideline databases and 74 from targeted searching and stakeholder outreach. After deduplication and screening of records, 72 clinical practice guidelines were included in the systematic review. Figure 1 presents the PRISMA flowchart of the guideline selection.

### Characteristics of included clinical practice guidelines

General characteristics of the 72 included clinical practice guidelines are shown in Table 2. Guidelines were from 42 different countries across 6 continents. Most included clinical practice guidelines were developed in Europe (*n* = 26) and Central and South America (*n* = 13), followed by Africa (*n* = 8). An additional seven guidelines were not nation-specific but international-based. The comprehensiveness of the dry mouth sections within the clinical practice guidelines varied from limited (*n* = 35; up to a few recommendations), moderate (*n* = 25; subsection or multiple recommendations) to extensive (*n* = 12; guideline, chapter or large subsection). For a detailed overview of each included clinical practice guideline, see Supplemental File 3.

**Table 2.** Characteristics of included clinical practice guidelines (*n* = 72).

Characteristics	Clinical practice guidelines *n* (%)
Geography	
Africa	8 (11.1)
Asia	7 (9.7)
Central & South America	13 (18.1)
Europe	26 (36.1)
North America	6 (8.3)
Oceania	5 (6.9)
International	7 (9.7)
Main topic	
Dry mouth	9 (12.5)
Oral care	8 (11.1)
Oral problems	3 (4.2)
Palliative care	47 (65.3)
Pharmacology	3 (4.2)
Others	2 (2.8)
Target patients	
Life-limiting illness (unspecified)	50 (69.4)
Cancer	20 (27.8)
Geriatric frailty	2 (2.8)
Comprehensiveness of dry mouth^a^	
Limited	35 (48.6)
Moderate	25 (34.7)
Extensive	12 (16.7)

a *Limited*: at least one, and up to a few recommendations. *Moderate:* subsection of guideline with multiple recommendations for assessment, oral care, treatment and/or patient education of dry mouth. *Extensive*: full guideline, chapter or large subsection providing recommendations on (almost all) facets of dry mouth treatment, including assessment, oral care, different management options and patient education.

### Quality appraisal

The quality appraisal per AGREE II for all included clinical practice guidelines and the rater reliability are presented in Supplemental File 4. Domain 4 (clarity and presentation) scored the highest, with 64/72 guidelines scoring ⩾60%. Domain 5 (applicability) scored lowest, with only 7/72 guidelines scoring ⩾60% and 45/72 scoring ⩽10%. Domain 3 (rigour of development) and domain 6 (editorial independence) also received low scores, both with only 15/72 guidelines scoring ⩾60%.

Two guidelines from Germany and the Netherlands were ‘recommended for use’ based on the AGREE II criteria,^51,52^ with another 14 clinical practice guidelines recommended with modifications. The clinical practice guideline from Germany recorded the highest overall score.

The rater reliability was consistently high for each domain of the AGREE II (ICC = 0.79–0.96, *p* < 0.001).

### Content analysis and synthesis

Four themes emerged from the synthesis: (1) assessment of dry mouth; (2) importance of oral care (3) management of dry mouth; and (4) patient education and family involvement. Full content analysis is presented in Table 3.

**Table 3.** Content analysis of dry mouth recommendations in the included clinical practice guidelines (*n* = 72).

Clinical practice guideline	Assessment	Oral Care	Management					Patient education & family involvement
	Medical history & oral exam		Treat cause	Substitutes	Stimulants	Medication	Dietary advice	
Albania (2012)^53^	• Review meds	• Good oral hygiene, moisten • End-of-life care instructions	• Adjust meds	• Water • Artificial substitutes	• Chewing gum • Vitamin C	• Pilocarpine		• Instruct oral care to patient • Involve family in oral care at end of life
Albania (2014)^54^		• Good oral hygiene		• Artificial substitutes				
Argentina (2004)^55^	• Assess oral health daily • Perform oral exam: ⩾1×/d	• Routine: clean 2×/d, moisten • Lips: cocoa butter, no Vaseline • If oxygen use: brush with baking soda 3×/d	• Humidify oxygen	• Chamomile	• Chewing gum, ice cubes • Acidic juice		• Increase fluid intake	• Instruct oral care to family
Argentina (2025)^56^	• Assess dry mouth several times a day	• Routine: moisten • Lips: Vaseline, cocoa butter						• Educate family on signs and treatment
Australia (2017)^57^		• Good oral hygiene • Rinse: 2 hourly mouthwash (baking soda) • Lips: Lanolin		• Water • Artificial: Biotene	• Tonic water, acidic juice, pineapple	• Pilocarpine: 4% 1–2 eye drops 3×/d		
Australia (2019)^58^		• Routine: clean ⩾2×/d, moisten • Avoid: chlorhexidine • Lips: lip balm, paraffin		• Water • Artificial substitutes	• Ice cubes • Acidic juice		• Increase fluid intake	• Involve family in oral care at end of life
Australia (2020)^59^		• Routine: clean frequently, moisten						
Belarus (2022)^60^		• Routine: clean after meals & before bed; regular rinsing		• Artificial substitutes• Iodine spray • Water	• Chewing gum, ice cubes	• Pilocarpine: 1% 5–10 eyedrops 3×/d	• Frequent meals • Avoid: acidic, spicy, bitter, salty; hot/cold; hard food	
Brazil (2012)^61^	• Perform oral exam	• Routine: clean ⩾3×/d; fluoride, moisten • Lips: lip balm, water-based lubricating gel • Avoid: Vaseline • End-of-life care instructions	• Manage disease • Avoid: alcohol, caffeine	• Water, fluids • Artificial substitutes; water-based lubricating gels (KY Jelly)	• Chewing gum, crushed ice • Pineapple, citrous fruits • Acupuncture	• Pilocarpine: eyedrops 4% 2–3 2×/d	• Increase fluid intake • Moisten foods • Avoid: spicy, dry, citrous, hot food	• Instruct oral care to patient/family • Involve family in oral care at end of life
Brazil (2023)^62^		• Routine: moisten frequently • Lips: any moisturiser		• Artificial substitutes				
Bulgaria (2019)^63^		• Routine: moisten frequently						
Cameroon (2021)^64^	• Perform oral exam regularly	• Routine: good oral hygiene & moisten • Lips: Vaseline, Bepanthene		• Water	• Chewing gum, candy, ice cubes • Pineapple, citrous fruits			
Canada (2017)^65^	• Assess for dry mouth symptoms • Perform oral exam daily • Tool: oral assessment guide	• Routine: standard; fluoride or xylitol products • Lips: water-based, pericare, before mouth care/meals/bed • End-of-life care instructions		• Water • Moisturising gels	• Ice chips		• Increase fluid intake • Avoid: acidic, spicy, sharp, hard food	• Instruct oral care to patient/family
Canada (2017)^66^		• Routine: 2–3×/d; fluoride • Rinse: water after eating	• Avoid: alcohol, tobacco	• Water • Artificial: Biotene	• Chewing gum	• Pilocarpine	• Avoid: sugary, acidic drinks	• Instruct oral care to patient • Educate patient on dental risks, functional impact • Patient guide
Canada (2019)^67^	• Review meds	• Routine: clean & moisten frequently • Rinse: after eating with water • Avoid: alcohol-based products, Vaseline • End-of-life care instructions	• Adjust meds • Manage disease • Humidifier • Avoid: alcohol, tobacco	• Water • Artificial substitutes, moisturising gels (e.g. Biotene)	• Chewing gum, lozenges	• Pilocarpine	• Avoid: acidic, sugary food	• Instruct oral care to patient • Patient guide
Canada (2019)^68^	• Conduct elaborate medical history (detailed instructions) • Perform elaborate oral exam (detailed instructions) • Review meds • Tool: numeric rating scale 0–10, Xerostomia grading scale (NCI CTCAE)	• Routine: clean 2–4×/d, rinse ⩾4×/d (saline, baking soda); Increase if DM worsens; fluoride • Avoid: chlorhexidine, alcohol-based hydrogen peroxide, iodine • Lips: water/aloe-based	• Adjust meds • Manage disease • Humidifier • Avoid: alcohol, caffeine, tobacco	• Water • Milk, butter, vegetable oil • Artificial substitutes, moisturising gels (e.g. Biotene, Moistir, Salivard)	• Chewing gum, ice cubes, popsicles, candy • Xylitol products • Acupuncture	• Pilocarpine: RT-only • Other: Cevimeline, Bethanechol, Amifostine	• Increase fluid intake: 2-2.5 l • Moisten & soften food • Papaya • Avoid: dry, acidic, sugar, hot food	• Instruct oral care, self-assessment, when to contact to patient/family • Patient guide
Canada (2021)^69^	• Conduct elaborate medical history (detailed instructions) • Perform oral exam • Review meds • Tool: numeric rating scale 0–10	• Routine: detailed instructions; clean after eating & before bed and Increase if DM worsens; moisten • Rinse: bland rinse (baking soda, salt) ⩾ 2×/d and Increase if DM worsens; chlorhexidine 0.12% if needed; fluoride • Avoid: glycerine, acidic, Vaseline • Lips: water/oil/lanolin-based • End-of-life care instructions	• Humidifier • Avoid: caffeine, alcohol	• Water • Artificial substitutes, moisturising gels	• Chewing gum, popsicles, ice cubes • Xylitol products • Acupuncture/TENS: RT-only	• Pilocarpine: RT-based, or palliative care • Bethanechol, Cevimeline: RT-only • Anethole trithione: palliative care	• Increase fluid intake • Frequent meals • Soft, mild taste; and enhance taste as preferred • Mildly acidic, cold fruits • Avoid: acidic, sugar, hot food	
Chile (2022)^70^	• Review meds • Perform oral exam	• Routine: brush, moisten • Rinse: mild, non-alcoholic • Lips, cocoa, no Vaseline		• Water • Natural lubricants (e.g. chamomile) • Artificial substitutes, moisturising gels every 2–3 h				• Instruct oral care to patient & family
Chile (2023)^71^		• Routine: clean & moisten frequently; assess hygiene daily • Lips: cocoa, no Vaseline		• Water	• Ice cubes		• Ice cream	• Involve family in oral care in end-of-life care
China (2023)^72^						• Pilocarpine: 5 mg 1 tablet 3×/d		
Colombia (2016)^73^		• Good oral hygiene		• Artificial substitutes	• Chewing gum, ice cubes • Pineapple	• Pilocarpine		• Involve family in oral care at end-of-life care
Costa Rica (2015)^74^		• Routine: moisten • Rinse: if ulceration • Lips: Vaseline	• Humidifier	• Artificial substitutes	• Chewing gum, ice cubes • Sour milk/ yoghurt/juice, sweets			
Denmark (2024)^75^	• Conduct medical history (causes, dehydration) • Perform oral exam (infection, dental) • Tool: PRO palliation, severity 1–4	• Good oral hygiene • Rinse: frequently; with sparkling water, chlorhexidine 0.1% • Fluoride, xylitol, glycerine products	• Manage disease	• Water • Artificial substitutes	• Chewing gum, ice cubes (with mint), lozenges with xylitol/ fluoride • Acidic foods, pineapple, sparkling water	• Pilocarpine: 4% 2–3 eyedrops 3×/d; not for RT-induced DM	• Increase fluid intake • Sage tea, thin oat soup	• Instruct oral care to patient • Involve family in oral care at end-of-life care • Patient guide
Denmark (2024)^76^	• Review meds • Tool: PRO palliation, severity 1–4	• Good oral hygiene • Lips: moisten • Rinse: chlorhexidine	• Adjust meds	• Artificial substitutes	• Ice cubes • Pineapple (frozen) • Xerodent lozenges (xylitol)	• Pilocarpine: 4% 2–4 eye drops 4×/d	• Increase fluid intake	
Ecuador (2014)^77^		• Good oral hygiene • Rinse: water with honey, or with thyme, Sage, chamomile, baking soda; mint water with baking soda		• Water • Artificial substitutes; recipe: water + methylcellulose + lemon essence	• Chewing gum, ice cubes • Pineapple, vitamin C 250 mg 4×/d		• Soft foods • Avoid: dry, spicy, acidic, bitter, strong flavours	• Instruct oral care and self-assessment to patient
Estonia (2021)^78^	• Assess causes • Tool: Integrated Palliative Outcome Scale (IPOS, 0–4)	• Good oral hygiene • Lips: moisten		• Water, fluids				
eSwatini (2011)^79^	• Perform oral exam regularly	• Routine: clean & moisten frequently • Rinse: baking soda or salt in water • Lips: petroleum jelly			• Ice cubes			• Educate on dry mouth & instruct oral care to patient/family
Ethiopia (2016)^80^	• Review meds • Perform oral exam regularly	• Routine: clean after each meal & at night; fluoride products • Lips: petroleum jelly or other • Rinse: after each meal & at night; saline, vinegar or lemon juice in water; antimicrobial: chlorhexidine gluconate 0.2%, baking soda in water.	• Manage disease • Adjust meds	• Water			• Increase fluid intake • Soft, moist foods • Avoid: sticky, dry, spicy, citrous, hot food	
Europe (2018)^81^	• Asses oral health at every assessment • Conduct medical history • Review meds • Perform oral exam at every assessment • Tool: Oral Health Assessment Tool (OHAT)	• Routine: clean ⩾2×/d; floss; fluoride • Lips: moisten • Rinse: chlorhexidine, or with essential oils	• Adjust meds • Avoid: smoking	• Water • Artificial substitutes, moisturising gels	• Chewing gum (xylitol)	• Pilocarpine • Cevimeline, physostigmine	• Avoid: acidic, sugary food	• Educate & instruct oral care to patient/family
Finland (2019)^82^		• Routine: clean 2×/d; moisten; fluoride • Lips: petroleum jelly		• Water • Natural: cooking oil • Artificial substitutes, moisturising gels	• Chewing gum (xylitol)	• Pilocarpine: RT-only		
Finland (2022)^83^	• Conduct medical history (impact eating, speaking; pain), ask daily • Review meds • Perform oral exam (how to instructions)	• Routine: clean 2×/d; fluoride • Rinse: after cleaning; saline or mild mouthwash • Lips: lip balm		• Water • Natural: cooking oil • Artificial substitutes; glycerol	• Chewing gum (xylitol), candies; crushed ice • Juice		• Avoid: sugary food	• Instruct oral care to patient/family
France (2022)^84^	• Conduct medical history (causes, pain, oral care routine); ask daily • Perform oral exam daily • Tool: oral assessment guide	• Routine: clean ⩾2×/d; after meals; swabs with sodium bicarbonate/ menthol • Lips: Vaseline (unless oxygen), lip balm • Rinse: bicarbonate 1.4% 3×/d • Avoid: glycerine • End-of-life care instructions		• Water • Cooking oil, butter • Artificial substitutes, moisturising gels (Hydral, BioXtra, Elgydium xeroleave, Artisial, Aequasyal)	• Ice cubes • Sour candies, fruit, sparkling water • Tongue massage		• Increase fluid intake; drinks preferred by patient	• Instruct oral care to patient/family
Germany (2020)^51^	• Assess regularly for dry mouth • Review meds • Tool: Integrated Palliative Outcome Scale (IPOS, 0–4)	• Routine: clean & moisten regularly • Lips: moisten • Avoid: glycerine		• Water, preferred flavours • Olive oil • Moisturising gels	• Chewing gum, ice cubes/chips • Pineapple, sour tea, sour candies		• Ice cream	• Patient guide
Hungary (2023)^85^		• Routine: moisten • Lips: Vaseline, lip cream • Rinse: if inflammations, with salt water and antibiotic rinses	• Humidifier	• Water	• Ice cube (with lemon)		• Increase fluid intake • More spices, sugar, vinegar, lemon; cold food; moist fruits • Avoid: hot, cold, spicy food	
India (2021)^86^	• Conduct elaborate medical history (detailed instructions) • Review meds • Perform oral exam	• Routine: clean ⩾2×/d, moisten, use rinse • Lips: water soluble, petroleum jelly • Rinse: water, saline, saline/baking soda, chlorhexidine or hydrogen peroxide	• Manage diseases • Humidify oxygen • Avoid: alcohol, tobacco	• Water • Artificial substitutes, e.g. E saliva spray, Duestom (glycerine 18%), • Moisturising gel	• Chewing gum, ice cubes • Pineapple, sour candies	• Pilocarpine:5 mg 3×/d, increase to 10 mg 3×/d if drug-induced or RT-induced DM	• Increase fluid intake	• Instruct oral care and self-assessment to patient
International (2010)^87^	• Examine regularly for dry mouth (and its complications) • Review meds	• Routine: clean ⩾2×/d; fluoride • Avoid: alcohol-based products	• Manage disease • Adjust meds	• Water • Artificial substitutes • Natural: butter, oil	• Chewing gum, sweets • Acupuncture, TENS • Avoid: acidic products	• Pilocarpine • (Bethanechol, yohimbine, anethol trithione)	• Avoid: sugary, acidic food	• Educate patient/family
International (2021)^88^				• Moisturising gels	• Chewing gum • Acidic candies • Acupuncture, TENS: RT-only	• Pilocarpine: RT-only • Bethanechol, cevimeline: RT-only		• Instruct oral care to patient • Educate on (dental) risks and functional, physical and social impact.
International (2022)^89^	• Assess regularly for dry mouth • Conduct elaborate medical history • Perform oral exam • Tool: Oral Symptom Assessment Scale (OSAS)	• Routine: clean ⩾2×/d; after each meal; moisten; rinse: water • Avoid: glycerine • End-of-life care instructions	• Manage disease • Adjust meds	• Water • Artificial substitutes	• Chewing gum • Avoid: pineapple, vitamin C, candies	• Pilocarpine • Bethanechol, cevimeline		• Involve family in oral care in end-of-life care
International (2024)^90^	• Conduct elaborate medical history • Perform oral exam (incl. salivary glands, saliva quantity/quality) • Tool: sialometry, visual analogue/ numeric rating scale, xerostomia questionnaires; NCI-CTCAE, RTOG/ EORTC, LENT-SOMA							
International (2024)^91^	• Review meds	• Good oral hygiene • Lips: lanolin/wax-based • Avoid: sodium lauryl sulphate, alcohol based, acidic	• Adjust meds • Humidifier	• Mucosal hydration device • Artificial substitutes	• Chewing gum, sweets • TENS • Massaging salivary glands	• Pilocarpine: tablet or eyedrops 5–10 mg 3–4×/d; max. 30 mg/d; rinse • Cevimeline: 30 mg 3×/d • Bethanechol: 10–25 mg 3×/d • Anethole trithione: 25 mg 3×/d	• Avoid: acidic, spicy food	
Italy (2024)^92^	• Perform oral exam ⩾2×/week	• Routine: clean & moisten frequently	• Humidifier • Adjust meds	• Water, fluids • Artificial substitutes (e.g. Xerotin)	• Chewing gum, crushed ice, popsicles • Pineapple	• Pilocarpine: 5 mg 3–4×/d		
Japan (2013)^93^	• Assess causes, distress by dry mouth • Review meds	• Routine: Good oral hygiene, moisten • Lips: petroleum jelly	• Manage disease • Adjust meds • Humidifier	• Water, fluids • Artificial substitutes, moisturising gel • Sesame/olive oil	• Chewing gum, ice chips, massaging jaw • Pineapple, Acidic fluids	• Cevimeline		• Instruct oral care to patient/family • Involve family in oral care in end-of-life care • Educate on dry mouth; communication example
Latin America (2004)^94^	• Conduct elaborate medical history (detailed instructions) • Review meds • Perform oral exam frequently • Tool: dry mouth severity grade 0–4; oral assessment guide	• Routine: good oral hygiene • Avoid: alcohol-based products • Lips: moisten	• Manage disease • Adjust meds • Avoid: alcohol, tobacco • Humidifier	• Water • Artificial substitutes	• Chewing gum, mint products, ice cubes, popsicles • Vitamin C, acidic sweets • Acupuncture	• Pilocarpine: 2.5 mg 3×/d • Bethanechol, methacholine, yohimbine	• Increase fluid intake: 8 glasses/day • Moisten food; use enzymes from: papaya, pineapple, ablandador de carne (meat tenderising spice) • Avoid: sugars, spicy, dry food	
Malaysia (2015)^95^		• Routine: Good oral hygiene, moisten • Lips: petroleum-based balm • Avoid: alcohol-based • End-of-life care instructions	• Manage disease • Adjust meds	• Water • Artificial: Biotin, Orazyme	• Pineapple	• Pilocarpine: 5–10 mg 3×/d		• Involve & instruct family in oral care in end-of-life care
Mexico (2017)^96^		• Good oral hygiene • Rinse: fluoride	• Humidifier • Avoid: alcohol, tobacco	• Natural lubricants: chamomile with lemon • Artificial substitutes	• Chewing gum, sweets, ice cubes	• Pilocarpine • Cevimeline	• Increase fluid intake: 2 l/day	• Instruct oral care to family
Mexico (2018)^97^	• Conduct medical history • Review meds • Perform oral exam	• Routine: clean ⩾3×/d, moisten • Lips: moisten	• Adjust meds	• Water, fluids • Natural products: rinse with chamomile + lemon • Artificial substitutes: recipe water + methylcellulose + lemon essence	• Chewing gum, candy, ice cubes • Pineapple, citrous fruits, vitamin C	• Pilocarpine: eye drops 4%	• Increase fluid intake	
Netherlands (2023)^98^		• Routine: moisten frequently • Rinse: saline, water • Lips: Vaseline • End-of-life care instructions		• Water • Natural: chamomile • Artificial: Biotene Oral Balance, BioXtra, Caphosol, Saliva Orthana • Avoid: glycerine	• Crushed ice, frozen fruit • Pineapple			• Involve family in oral care in end-of-life care • Educate family on dry mouth
Netherlands (2025)^52^	• Assess dry mouth at every consultation • Conduct elaborate medical history (detailed instructions) • Differentiate dry mouth and salivary gland hypofunction • Review meds • Perform oral exam at all physical examinations (detailed instructions) • Tool: shortened Xerostomia Inventory (s-XI); numeric rating scale 0–10; oral health assessment tool; CODS; sialometry; GOHAI.	• Routine: clean 3–4×/d, fluoride; moisten frequently • Rinse: saline • Lips: moisturising balm • Avoid: alcohol-based; Vaseline • End-of-life care instructions	• Manage disease • Adjust meds • Reduce: tobacco, caffeine • Humidifier	• Water • Artificial substitutes • Natural: salvia officinalis (Sage)	• Chewing gum (xylitol), fruits, crushed ice • Pineapple • Acupuncture, TENS	• Pilocarpine: 3×/d 2.5–5mg if SGH	• Increase fluid intake • Frequent meals • Moisten food • Avoid: spicy, dry, hard, sugary food, citric fruits	• Educate patient/family on dry mouth • Instruct oral care to patient/family • Patient guide & resources
New Zealand (2020)^99^	• Assess causes, emotional/social impact • Review meds • Perform oral exam • Tool: Integrated Palliative Outcome Scale (IPOS, 0–4)	• Good oral hygiene • Rinse: salt/baking soda, chlorhexidine (depending on cause) • End-of-life care instructions	• Manage disease	• Moisturising gels	• Ice chips • Pineapple, citrous fruits	• Pilocarpine: 1 mg/ml, 5 ml 3×/d	• Increase fluid intake	
New Zealand (2024)^100^	• Conduct elaborate medical history (detailed instructions) • Review meds • Perform oral exam daily	• Routine: clean ⩾2×/d	• Reduce: caffeine, alcohol	• Saliva substitutes	• Chewing gum (xylitol), popsicles • Pineapple, acidic juice, melon	• Pilocarpine”1 mg/ml 5–10 ml or 1–2 drops 4% 3×/d		
Norway (2019)^101^	• Conduct medical history • Review meds • Perform oral exam • Tool: numeric rating scale 0–10; sialometry; spatula test	• Routine: Good oral hygiene; use fluoride daily • Rinse: saline; sodium bicarbonate; calcium/phosphate • Avoid: foaming agents (sodium lauryl sulphate), chlorhexidine	• Adjust meds • Avoid: alcohol, tobacco	• Water, fluids • Artificial substitutes	• Chewing gum (xylitol), lozenges (acidic, fluoride, xylitol) • Vitamin C + acetylcysteine in case of thick saliva	• Pilocarpine (not registered in Norway)	• Increase fluid intake • Soft food, frequent meals • Avoid: sugar, spicy dry	• Educate on dry mouth & instruct oral care to patient/family • Patient guide
Portugal (2021)^102^	• Review meds	• Routine: optimise oral hygiene • Lip: lubricate	• Adjust meds	• Water • Artificial substitutes	• Chewing gum, sour sweets, ice cubes • Pineapple, cucumber, apple (frozen)	• Pilocarpine: 5 mg tablets 3×/d; 15 eye drops 3×/d • Cevimeline: 30 mg 3×/d	• Increase fluid intake • Soft, moist, cold food	
Saudi Arabia (2019)^103^	• Conduct medical history • Review meds • Perform oral exam	• Routine: clean well ⩾1×/d, moisten • Rinse: Magic Mouth Wash 5 ml 3×/d • Lips: lubricate	• Avoid: alcohol, caffeine	• Water • Artificial substitutes	• Chewing gum (xylitol), popsicles, ice cubes • Lightly acidic fruits, cold cucumber/ tomato/apple • Acupuncture: RT-only	• Pilocarpine: 5 mg 3×/d	• Increase fluid intake • Soft, moist, mild tasting, cold food • Milk, jelly, sherbet, applesauce, ice cream • Avoid: acidic, sugar, dry food	• Educate patient/family on dry mouth, risks, treatment • Instruct oral care to patient/family
Scotland (2024)^104^	• Assess causes • Review meds • Perform oral exam	• Routine: clean ⩾4×/d, fluoride toothpaste after each meal; fluoride rinse daily • Rinse: saline • Lips: water-based • Avoid: petroleum jelly • End-of-life care instructions	• Manage disease • Adjust meds • Humidify oxygen • Humidifier	• Water • Artificial substitutes	• Chewing gum, ice chips, sweets • Tonic water		• Increase fluid intake • Reduce: sugary food	• Involve & instruct family in oral care in end-of-life care • Patient guide
South Africa (2012)^105^	• Check for oral symptoms regularly • Assess causes • Review meds • Perform oral exam at each visit • Tool: oral assessment guide	• Routine: clean & moisten 2–4×/d, if severe 8–12×/d; assess daily; use fluoride • Rinse: water, saline, saline-sodium bicarbonate • Lips: Vaseline • Avoid: glycerine, hydrogen peroxide • End-of-life care instructions	• Manage disease • Adjust meds • Avoid: alcohol, caffeine, tobacco • Humidifier • Humidify oxygen	• Water • Natural: margarine, salad oil before eating • Artificial: water-soluble (KY jelly); Betaine, Biotene, Xerostom	• Chewing gum, ice chips • Pineapple, acidic sweets, diluted fruit juice, tonic water, vitamin C	• Pilocarpine: eyedrops 4% 2–3 drops p.o. 3×/d • Bethanechol: 10 mg 3×/d	• Increase fluid intake • Moisten food • Avoid: dry, sticky food	• Educate on dry mouth • Instruct self-assessment and oral care to patient/family
Spain (2008)^106^	• Regular self-examination by patient	• Routine: clean regularly • Avoid: alcohol, lemon, glycerine		• Artificial substitutes	• Chewing gum, ice cubes • Pineapple	• Pilocarpine: 5–10 mg 3×/d		• Instruct self-assessment and oral care to patient
Spain (2021)^107^		• Routine: clean well, moisten • Lip: lip balm, cocoa • Avoid: Vaseline		• Water, fluids	• Ice cream, juices, acidic fruits (e.g. pineapple), chamomile with lemon			• Patient guide
Spain (2021)^108^		• Good oral hygiene		• Water • Artificial substitutes	• Chewing gum, candies; ice cubes • Pineapple	• Pilocarpine: 5–10 mg 3×/d	• Increase fluid intake	
Spain 7 (2023)^109^	• Review meds	• Good oral hygiene • Routine: rinse & brush after meals + before bed • Rinse: water; povidone/water, hydrogen peroxide/water	• Treat disease • Adjust meds • Avoid: alcohol, caffeine, tobacco • Humidifier	• Water, fluids • Natural: chamomile • Artificial substitutes	• Chewing gum, candies, ice • Pineapple • Acupuncture	• Pilocarpine: 1–2 eyedrops 3×/d; tablet 5–10 mg 3×/d	• Increase fluid intake; lemon juice • Avoid: sugary food	• Encourage oral care
Sri Lanka (2021)^110^	• Review meds • Perform oral exam	• Routine: ⩾ 4×/d • Lips: water-based gel	• Adjust meds	• Water • Avoid: acidic product, e.g. Glandosane	• Chewing gum, crushed ice, sweets • Pineapple		• Increase fluid intake • Avoid: sugar, carbonated drinks, juices	
Uganda (2012)^111^		• Good oral hygiene		• Water, fluids	• Ice cubes • Pineapple, acidic fruits			• Educate family on dry mouth
Uganda (2023)^112^	• Review meds	• Routine: clean ⩾3×/d • Rinse: salted water hourly • Lips: Vaseline • End-of-life care instructions			• Pineapple		• Soft food • Avoid: sugary food	
Uganda (unknown)^113^	• Screen regularly • Conduct medical history • Review meds • Perform oral exam	• Routine: clean ⩾2×/d • Rinse: saline or bicarbonate • Lips: lip balm, Vaseline • End-of-life care instructions	• Adjust meds	• Water, fluids	• Pineapple		• Soft, moisten food • Avoid: sugary food	
United Kingdom (2015)^114^		• Routine: frequent mouth care	• Adjust meds	• Water, fluids				• Involve & instruct family in oral care at end-of-life care
United Kingdom (2018)^115^		• Routine: good oral hygiene; fluoride • Lips: petroleum jelly, emollients	• Avoid: alcohol, tobacco	• Water • Artificial: Saliva Orthana, Biotene Oral Balance, BioXtra • Avoid: acidic products (Glandosane), if dentate • Acupuncture	• Chewing gum	• Pilocarpine: RT-only; 5–10 mg 3×/d	• Avoid: hard, spicy, acidic food; fizzy, acidic drinks	
United Kingdom (2019)^116^	• Assess causes, pain, fatigue, nutrition • Review meds • Tool: oral assessment guide, visual analogue scale, NCI-CTCAE	• Good oral hygiene • Rinse: saline • Lips: Vaseline; if RT or oxygen: water-soluble lubricants		• Water • Artificial: Caphosol, Xerotin	• Chewing gum, pineapple	• Pilocarpine: RT-only	• Increase fluid intake	• Instruct self-assessment to patient
United Kingdom (2023)^117^	• Conduct elaborate medical history (detailed instructions) • Review meds • Perform oral exam (detailed instructions)	• Routine: brush 2×/d, increase if high risk, fluoride, floss, • Rinse: warm water after each meal and at night; chlorhexidine if furred tongue • Lips: petroleum jelly, water-soluble • End-of-life care instructions	• Manage disease • Adjust meds • Humidify oxygen • Humidifier	• Water • Artificial substitutes • Avoid: acidic products (e.g. Glandosane) if dentate	• Chewing gum, sweets, ice chips • Avoid: acidic foods if dentate; may consider pineapple		• Increase fluid intake	
United Republic of Tanzania (2020)^118^	• Assess causes • Review meds	• Good oral hygiene	• Avoid: caffeine	• Water, fluids • Artificial: Biotene Oral Balance, AS Saliva Orthana	• Chewing gum, sweets, frozen fruit, pineapple		• Increase fluid intake • Soft, moist food	
United States of America (2016)^119^	• Conduct elaborate medical history • Review meds • Perform oral exam (incl. palpation salivary glands) • Tool: sialometry	• Good oral hygiene • Routine: fluoride • Rinse: saline, sodium bicarbonate		• Water • Artificial substitutes	• Chewing and taste; e.g. chewing gum, lozenges • Acupuncture, (T)ENS • Avoid: acidic products if dentate	• Pilocarpine • Cevimeline		• Educate patient on dry mouth treatment options
Uruguay (2017)^120^	• Question & perform oral exam daily	• Routine: clean after each meal or ⩾ 3×/d, moisten • Lips: Vaseline, cocoa butter • Avoid: xylitol • End-of-life care instructions		• Water	• Chewing gum, ice cubes • Citrous, vitamin C		• Increase fluid intake	• Instruct oral care to patient
Venezuela (2012)^121^	• Conduct medical history • Perform oral exam, quantify saliva production	• Routine: clean after meals and at night; fluoride • Rinse: chlorhexidine after eating • Lips: cocoa butter	• Humidifier	• Water • Natural: vegetable oil • Artificial substitutes	• Sour chewing gum, citrous candies, fruit	• Pilocarpine: 5–10 mg 3×/d (unavailable in Venezuela)	• Increase fluid intake; ⩾ 1.5 l d, water, citrous juices • Soft, moist food • Avoid: sugary, dry, spicy, acidic food	• Educate patient and family
Vietnam (2022)^122^	• Tool: Vietnamese Palliative Outcome Scale (VietPOS, 0–5)			• Water				

Empty cell means: no recommendation available; NCI-CTCAE: NCI common terminology criteria for adverse events; RTOG/EORTC: radiation therapy oncology group/European organisation for research and treatment of cancer; LENT-SOMA: late effects normal tissue task force-subjective, objective, management, analytic; PRO Palliation: Danish patient-reported outcome form for palliation, CODS: clinical oral dryness score, GOHAI: geriatric oral health assessment index; meds: medication; (*n*)×/d: *n* times a day, Medication: RT-only: only indicated for radiotherapy-induced dry mouth; Stimulants: (T)ENS: (transcutaneous) electrical nerve stimulation; end-of-life care: care in the final days or weeks before death.

## Theme 1: Assessment of dry mouth

### Medical history (n = 32)

Structured and detailed questioning about dry mouth symptoms was recommended in 32 clinical practice guidelines. Topics deemed important for assessment included:

1. Information on the complaint (presence, severity, frequency, duration, fluctuation);
2. The impact of dry mouth symptoms on daily functioning;
3. Potential causes, including a review of medical history, medication and recent interventions, dietary habits and intake, history of smoking and alcohol, and oral hygiene
4. Factors that may influence the choice of intervention (such as comorbidities or the prognosis);
5. The oral care routine, and the ability to carry out oral care independently.

The most frequent recommendation was the review of medication to identify any drugs with possible xerostomic effects (*n* = 31). While most guidelines mentioned the functional and physical impact of dry mouth symptoms (such as the impact on nutritional status), only eight guidelines recommended asking about the psychosocial impact of dry mouth and coping ability.^69,81,86,89,90,94,99,117^

### Oral examination (n = 36)

Oral examination to evaluate the severity of dry mouth and oral health status was recommended in 36 guidelines. Specific recommendations focussed on the presence of dry, cracked lips, the amount and texture of saliva, tongue fissures, the presence of halitosis, signs that may indicate concurrent infection (fungal, viral or bacterial), mucositis, gingivitis/periodontitis, and ill-fitting dentures.

### Regular monitoring (n = 23)

Specific frequency for monitoring of dry mouth was recommended in 23 guidelines: daily assessment (*n* = 8),^55,56,65,83,84,100,105,120^ at least twice a week (*n* = 1),^92^ and at every contact with a health care provider (*n* = 5),^52,68,88,105,116^ while other guidelines gave less detailed instructions (*n* = 9, ‘often’, ‘regularly’).^51,66,87,89,94,105,113,116,119^

### Measurement instruments (n = 19)

Specific measuring instruments were recommended in 19 guidelines. These were recommended for use in assessment and monitoring of dry mouth severity, of frequency and impact, or in the differentiation between xerostomia and salivary gland hypofunction.

Patient-reported outcome measures focussed on severity or impact on quality of life, and included numeric rating scales (NRS) ranging from 0 to 10 (*n* = 5),^52,68,69,90,101^ 1–4 (*n* = 2),^75,76^ and 0–4 (*n* = 4),^51,78,99,122^ the shortened Xerostomic Inventory (s-XI, *n* = 2),^52,90^ and oral health assessment tools (*n* = 2; OSAS; EORTC QLQ - OH15).^89^ Physician initiated tools included oral assessment guides (*n* = 7, of which *n* = 2 for the validated Oral Health Assessment Tool (OHAT)),^52,65,81,84,94,105,116^ the spatula/mirror test (*n* = 1),^101^ and dry mouth severity grading scales (Xerostomia NCI CTCAE grading scale (*n* = 3) and other (*n* = 2)).^52,68,90,94,116^ Three guidelines recommended sialometry to differentiate between xerostomia and salivary gland hypofunction/hyposalivation.^52,101,119^ One guideline noted that sialometry might be inappropriate for some patients with a life-limiting disease due to added burden.^52^

## Theme 2: Oral care

### Importance of oral care (n = 68)

68 clinical practice guidelines emphasised the importance of oral care within the prevention and management of dry mouth.

### Oral care routine (n = 60)

Specific oral care instructions included: brushing teeth a minimum of twice a day (*n* = 25), using a mild toothpaste with fluoride (*n* = 22), or a prescription-strength fluoride toothpaste (5000 ppm; *n* = 7)^52,68,69,81,87,89,115^; using a mild, non-alcoholic mouth rinse (*n* = 50; water, saline, baking soda and/or fluoride); and avoiding alcohol-based oral care products (*n* = 13).

Moisturising the lips was recommended by 47 clinical practice guidelines, with petroleum jelly (e.g. Vaseline; *n* = 18), water-soluble lubricants (*n* = 12) and cocoa butter (*n* = 7)^55,56,70,71,107,120,121^ as the most recommended products. Six clinical practice guidelines specifically recommended against using petroleum jelly due its hydrophobic and flammable nature.^52,55,61,104,107,116^ Glycerine-based toothpaste and moisturising gels were recommended in three^75,86,107^ but cautioned against in seven guidelines,^51,52,69,84,89,98,105^ due to reported humectant benefits versus potential dehydrating effects.

### Oral care in the weakened or dying person (n = 22)

Recommendations specific to the weakened or dying person were offered in 22 guidelines. These focussed on oral comfort rather than oral hygiene, increasing the frequency of oral care and moistening the mouth with soft materials other than toothbrushes (like swabs, gauzes, sponge sticks) and with sprays.

### Role of the dental health care professional (n = 22)

Active involvement of a dental professional was recommended in 22 guidelines, for regular checkups, and for specialist advice for treatment-resistant dry mouth and concurrent oral problems.

## Theme 3: Management of dry mouth

Recommendations for the management of dry mouth mostly consisted of treating the underlying cause, (non)-pharmacological saliva substitution, non-pharmacological saliva stimulation, dietary advice and medication.

### Treating underlying cause (n = 40)

Interventions treating the underlying cause included replacing or removing xerogenic medication (*n* = 26); managing underlying diseases (e.g. diabetes, HIV infection, Sjogren’s syndrome; *n* = 19); treating oral infections and diseases (*n* = 11), humidifying patients’ oxygen (*n* = 6)^55,67,86,104,105,117^; using a room humidifier in dry or warm environments (*n* = 16); and reducing or avoiding smoking (*n* = 12), alcohol use (*n* = 14) and caffeine consumption (*n* = 9).^52,61,68,69,100,103,105,109,118^

### Substituting saliva (n = 66)

Frequently sipping fluids, or using sponges/swabs with fluids, was recommended in 55 clinical practice guidelines to substitute saliva and moisten the mouth. Most clinical practice guidelines recommended water or unsweetened, non-acidic drinks to moisten the mouth, while four mentioned to offer any drinks the patient prefers. Saliva substitution products made from natural or household compounds were recommended (*n* = 24), including cooking oil (*n* = 11), chamomile (*n* = 8),^70,55,77,96–98,107,108^ and sage (*n* = 2).^52,77^

Commercial saliva substitutes to alleviate dry mouth were recommended in 53 guidelines. Common substitutes included: Biotene (OralBalance) spray or gel (*n* = 11), AS Saliva Orthana (*n* = 6)^52,53,98,104,115,118^ and BioXtra gel or spray (*n* = 3).^84,98,115^ The most common active ingredients in these substitutes were carboxymethylcellulose or hydroxyethyl cellulose, mucin, glycerine, xylitol, and fluoride. Half of the 12 guidelines that recommended a mucin-based product also mentioned its porcine origin, and potential issues with cultural, religious or dietary beliefs. Four clinical practice guidelines mentioned a cost-effective and accessible alternative for commercial saliva substitute: intraoral use of a water-based lubricating gel, primarily used for lubrication during intimacy (K-Y Jelly),^62,105^ and a recipe to make artificial saliva with methylcellulose, lemon essence and water.^77,97^

### Stimulating saliva (n = 62)

#### Mechanical and gustative saliva stimulants (n = 62)

Frequently recommended mechanical stimulants were chewing on (sugar-free) gum (*n* = 44) and sucking on ice cubes, crushed ice or frozen pieces of fruit (*n* = 42). Other examples of mechanical stimulants were chewing on (sugar-free) hard sweets (*n* = 11) and sucking on pieces of fruit, lollipops and lozenges (*n* = 21).

The most mentioned gustative stimulants were acidic products, such as citrous fruits, sour sweets and fruit juices (*n* = 57). However, 22 clinical practice guidelines recommended avoiding these acidic products, due to their erosive and ulcerative effect on teeth and oral mucosa. Clinical practice guidelines (*n* = 36) that did recommend the use of (lightly) acidic products to stimulate saliva, specifically mentioned fresh or frozen pineapple (*n* = 31), sour candies (*n* = 7),^51,74,84,86,88,102,105^ vitamin C lozenges or tablets (*n* = 8),^53,77,89,94,97,101,105,120^ and sour teas (*n* = 3).^51,96,101^ Pineapple was also mentioned to be beneficial for dry mouth due to the soothing effect of its anti-inflammatory enzyme bromelain on oral mucosa (*n* = 3).^51,105,117^

#### Other saliva stimulants (n = 10)

Acupuncture was recommended in nine guidelines in the context of saliva stimulation (*n* = 7 for all patients with a life-limiting disease or frailty^52,68,87,94,109,115,119^; *n* = 3 for patients with radiotherapy-induced xerostomia only^69,88,103^).

Other saliva stimulants recommended were xylitol-based products (*n* = 12); sparkling or tonic water (*n* = 5),^57,75,84,104,105^ (transcutaneous) electrostimulation (recommended for all patients *n* = 4^52,87,91,119^; for radiotherapy-induced xerostomia only *n* = 2^69,88^); massaging the tongue, the oral cavity, the jaw and/or the parotid and submandibular gland (*n* = 3).^84,91,93^

#### Dietary advice (n = 45)

Dietary advice consisted of the amount of fluids, and the type of food and fluids that are appropriate and pleasant for patients with dry mouth.

Adequate fluid intake was recommended in 30 clinical practice guidelines, with four guidelines providing extra instructions for the amount of fluid, ranging from 1.5 to 2.5 l per day.^68,94,96,121^

Dietary recommendations were provided in 35 clinical practice guidelines. These included eating moist food or moistening food (*n* = 19), foods with soft consistency (*n* = 15), and eating frequently with small bites (*n* = 6).^52,60,65,68,69,118^ Particular foods were recommended to be avoided including dry and hard food (*n* = 13), spicy food (*n* = 13), thermally aggressive food (*n* = 8),^60,61,68,69,80,85,102,103^ sugary food (*n* = 20) or acidic food (*n* = 12). Few guidelines gave recommendations for specific food items or dishes. A Danish clinical practice guideline recommended sage tea and thin oat soup *(thynd havresuppe)* to alleviate sore mucosa due to dry mouth,^75^ and three other guidelines recommended papaya.^68,77,94^

### Medication (n = 40)

The pharmacological interventions most mentioned included pilocarpine (*n* = 37), bethanechol (*n* = 8), and cevimeline (*n* = 10).

#### Pilocarpine (n = 37)

Pilocarpine was recommended in 37 guidelines, with no consensus on dosage, form or frequency:

- Pilocarpine eye drops (off-label; *n* = 9)^57,60,75,76,91,97,100,102,105^: pilocarpine 4% with dose ranging from 1 (~2 mg) to 5 (~10 mg) drops 3×/day; pilocarpine 1% with dose ranging from 5 (~2.5 mg) to 20 drops (~10 mg) 3×/day.
- Pilocarpine tablets (*n* = 13)^52,72,86,90–92,94,95,102,103,106,108,109^: dose ranging from 2.5 to 10 mg 3×/day.
- Pilocarpine rinse (*n* = 2)^99,100^: pilocarpine 1 mg/ml with a dose of 5–10 ml (5–10 mg) 3×/day.

32 clinical practice guidelines recommended pilocarpine for all patients with dry mouth, while five clinical practice guidelines recommended pilocarpine for radiotherapy-induced dry mouth only.^68,82,88,115,116^

#### Bethanechol (n = 8) and Cevimeline (n = 10)

Bethanechol as a pharmacological saliva stimulant was mentioned in eight clinical practice guidelines.^68,69,87–89,91,94,105^ Advice on dosage was provided in two guidelines: 10 mg 3×/day or 10–25 mg 3×/day.^91,105^ Cevimeline was mentioned in 10 clinical practice guidelines,^68,69,81,88,89,91,93,96,102,119^ of which two advised on dosage: 30 mg 3×/day.^91,102^ Cevimeline and bethanechol were only recommended for radiotherapy-induced xerostomia in two guidelines.^69,88^

#### Other medication

Other dry mouth medication mentioned were: anethole trithione (*n* = 3),^69,87,91^ local application of physostigmine (*n* = 1),^81^ methacholine (*n* = 1)^94^ and yohimbine (*n* = 2).^87,94^

### Collaboration and referral (n=12)

Several guidelines (*n* = 12) emphasised the need for strong collaboration between healthcare professionals. This included seeking additional support from dieticians/nutritionists and speech and language therapists.

## Theme 4: Patient education and family involvement (n = 43)

Most guidelines (*n* = 43) recommended educating patients and family on the importance of oral care and providing specific instructions. Other recommendations focussed on providing information on dry mouth, its complications and its treatment; answers to questions on the thirst versus dry mouth debate in end-of-life care; instructions to self-assess and monitor dry mouth regularly; and advice for substituting and stimulating saliva. One guideline provided written-out conversation guides for patient education.^93^

Specific resources such as brochures or websites were recommended in 10 clinical practice guidelines.^51,52,66–68,75,101,104,107,115^ These corresponded with the recommendations provided in the clinical practice guidelines for professionals.

In the context of the weakened or dying patient, 26 clinical practice guidelines specifically recommended to actively involve the family in providing oral care, such as cleaning and lubricating the mouth and lips. It was mentioned that this can provide the family with a sense of usefulness and an active role in comforting their loved one.^71,93,98^ However, as some might find it too distressing or difficult, one guideline warned to not force the family to be involved in mouth care.^89^

## Discussion

This systematic review analysed the quality and content of clinical practice guidelines for dry mouth in palliative care. 72 guidelines across 42 countries were identified. Comprehensiveness of dry mouth specific recommendations was limited, with only 12 guidelines having a major focus on dry mouth. Methodological quality of the clinical practice guidelines was low to moderate, and the evidence base underlying the recommendations was limited. However, recommendations were largely consistent for the four main themes of dry mouth care practices: the assessment of dry mouth, the importance of oral care, the management of dry mouth, and patient education and family involvement (see Table 4).

**Table 4.** Consensus of recommendations for dry mouth in palliative care.

Theme	Recommendations
Assessment of dry mouth	• Dry mouth and other oral problems should be assessed regularly in patients with life-limiting illnesses. • An assessment should include a detailed medical history, a review of medication and an oral examination.
Oral care	• Good oral hygiene is important for the prevention and treatment of dry mouth. • Oral care routines for dry mouth include brushing teeth ⩾2 times a day, and using alcohol-free, fluoride-based oral care products.
Management of dry mouth	• Treat the cause of dry mouth, if possible. • Alleviate dry mouth by: ○ Moistening the mouth frequently, with water and other natural lubricants or with artificial saliva substitutes. ○ Stimulating saliva production with mechanical and gustative stimulants, including chewing on chewing gum and sucking on ice cubes. ○ Adapting the diet and increasing fluid intake (if appropriate), moistening the food and avoiding sugary, spicy and dry foods. ○ Considering prescribing pilocarpine.
Patient education & family involvement	• Educate patients and their relatives on the importance of oral care and provide oral care instructions. • Offer to involve relatives in providing oral care for their weakened or dying loved one, as this may provide a sense of usefulness.

Our review shows a lack of robust clinical guidance for dry mouth in palliative care, with only two guidelines being ‘recommended for use’ based on current quality.^51,52^ The AGREE II domains *Rigour of Development* and *Applicability* scored particularly poorly, largely due to a lack of information on search strategy and evidence grading, and of implementation strategies. Contributing factors for low quality of the clinical practice guidelines include a scarcity of scientific evidence in dry mouth and palliative care research,^30–32^ and palliative care being a relatively new and evolving practice, with limited funding in certain geographic regions.^38,44–47^ Interestingly, methodological quality did not seem to influence content or clinical validity of recommendations across clinical practice guidelines. For example, clinical practice guidelines with higher and lower methodological quality (e.g. Netherlands^52^ and Germany^51^ vs France^84^ and Cameroon,^64^ respectively) all provided consistent and appropriate recommendations for dry mouth care, such as similar oral care instructions and saliva substitutes and stimulants. This is in line with previous research suggesting that the methodological quality of clinical practice guidelines is not directly associated with the content or the clinical validity of the recommendations.^35,123,124^

Consistency of recommendations also applied across country and economic status, with few guidelines addressing local practices or adapting recommendations to the specific context. For example, some guidelines recommended medication unavailable within their region (e.g. cevimeline is unavailable in Europe)^102,125^ and most did not provide information on the cost of different treatment options (e.g. artificial saliva products or dental care are uncompensated care in many countries). While it is encouraging that most guidelines share similar best practices, the uptake of clinical practice guidelines could be improved by adapting recommendations to the local context.^126^

Few guidelines gave instructions for clinicians to conduct a structured assessment of dry mouth through anamnesis or oral examination. Guidelines lacked detail on the frequency of assessment, advice on which questions to ask, and consensus on assessment instruments. Assessment is a critical step for adequate recognition and treatment of dry mouth,^15,127^ acting early to manage symptoms when they develop, acknowledging the severity of symptoms and being aware of potential contributing factors. Given that many healthcare professionals lack confidence in conducting these assessments,^25,26^ this is a key area where implementation of guidelines into the education of clinicians has potential to increase awareness and help the management of dry mouth.

There was a lack of clear consensus for pharmaceutical interventions for dry mouth in palliative care, in particular for the use of pilocarpine. This included different recommendations on the use of pilocarpine in specific patient groups (i.e. only for radiotherapy-induced or all cases of dry mouth), on dosage and form. These inconsistencies likely stem from a lack of high-quality scientific evidence for pharmacological interventions for dry mouth in palliative care, including pilocarpine.^31,32^ To illustrate this, a recent scoping review of Kakei et al. (2024) revealed only two intervention trials in the palliative care setting.^31^ Both trials showed limited effectiveness with moderate to severe side effects. Latest pilocarpine research has focussed on feasibility trials with topical forms of pilocarpine to retain the effectiveness but limit the side effects.^128–130^ Building this evidence base for the effectiveness and recommended use of pilocarpine should continue to be a priority in palliative care research.

It was notable that little emphasis was placed in the guidelines on managing the psychosocial distress induced by dry mouth. Only eight guidelines mention prompts about quality of life during assessment of dry mouth^69,81,86,89,90,94,99,117^ and one guideline as part of patient education.^88^ This is not surprising as both patients and health care professionals have previously reported a lack in acknowledgement of the dry mouth symptom itself as well as the negative impact on quality of life by dry mouth.^19,26^ However, dry mouth can severely interfere with speaking, eating, intimacy and sleeping^20^ – arguably activities that are specifically important for patients and their loved ones in the palliative care setting. By not addressing dry mouth and its physical and psychosocial consequences, health care professionals may unknowingly contribute to its high symptom burden. By incorporating all dimensions of palliative care (physical, psychological, social and spiritual)^38^ in guidelines for dry mouth, it may increase the perceived importance of addressing dry mouth in all its facets.

### Strengths and limitations

A key strength of this systematic review is the comprehensive search strategy, which combined conventional search methods in scientific databases with grey literature searches and a stakeholder outreach campaign to enhance scope and inclusivity of findings.^40^ With a broad definition of clinical practice guidelines, no restrictions on type of life-limiting illness, and no language restriction, the review achieved extensive geographic representation, covering six continents, and ensuring greater inclusion of low- and middle-income countries.

Despite this, the review cannot be seen as exhaustive as some clinical practice guidelines may still not have been identified due to accessibility barriers, a country’s use of local resources rather than national guidelines or missing newly published guidelines. In addition, the extent to which the identified clinical practice guidelines are implemented in practice remains unknown. While efforts were made to verify the use of clinical practice guidelines with palliative care stakeholders, confirmation was not possible for all guidelines (see Supplemental File 3). Finally, the absence of restrictions on illness type may limit the specificity of recommendations for some patient groups, as the aetiology and contributing factors of dry mouth vary across patient populations.

### Implications for future research & clinical practice

This review demonstrated that most guideline recommendations for dry mouth care in palliative care are based on expert opinion or low-grade scientific evidence. This is in line with previous systematic reviews on interventions for dry mouth.^31,32^ Future research should therefore focus on evaluating current and new dry mouth care practices within the palliative care setting in robust clinical trials. Future research priorities should include comparing effectivity and acceptability between different saliva substitutes and stimulants, and researching optimal pharmacological treatment options (including pilocarpine and bethanechol). These studies should also assess the impact on dry mouth induced psychosocial distress.

This review highlights the need for guidelines that provide clear and specific recommendations addressing all relevant components of dry mouth assessment and management in palliative care. Improving clarity of recommendations is specifically important, as the lack of clear guidelines has been mentioned previously by health care professionals as an important barrier for the treatment of oral health problems in palliative care.^23,26^ Clear and easy-to-follow instructions have been marked as an important attribute for the uptake of guidelines by healthcare professionals.^131^ Future guidelines should therefore include detailed instructions for the assessment, treatment and monitoring of dry mouth, such as *when and how* to use a particular treatment or *how* and *how often* to assess dry mouth. As goals of care and priorities for dry mouth management may differ across palliative care phases, including the disease-modifying, symptom-directed and terminal phase, guidelines should also consider phase-specific recommendations.^132^ In addition, they should address the (psychosocial) impact on quality of life of patients.^18,22^ Most importantly, future efforts should prioritise the effective implementation of existing and new guidelines. This requires practical, user-friendly strategies, such as concise summaries and digital decision-support tools, and recommendations that are adapted to cultural and socio-economic contexts and to the specific characteristics of patient populations.

## Conclusion

This systematic review analysed the quality and content of clinical practice guidelines for dry mouth in palliative care. The review found that the comprehensiveness of dry mouth recommendations was limited, the methodological quality of the clinical practice guidelines was low to moderate, and the evidence base underlying the recommendations was lacking. Despite this, many care practices appear to be shared worldwide, including oral care, saliva substitutes and stimulants, dietary advice and the use of pilocarpine. This review highlights the need for higher quality, easy-to-use guidelines that comprehensively focus on dry mouth in palliative care, and are effectively implemented in practice. Underpinning these guidelines is the need for future research evaluating treatment interventions specifically within the palliative care setting. These steps can make important contributions towards improving the quality of care for people with life-limiting illnesses suffering from dry mouth.

## Supplemental Material

sj-docx-1-pmj-10.1177_02692163261434188 – Supplemental material for Dry mouth in palliative care: A systematic review of clinical practice guidelines around the world Supplemental material, sj-docx-1-pmj-10.1177_02692163261434188 for Dry mouth in palliative care: A systematic review of clinical practice guidelines around the world by A. I. van der Meulen, A. Stoppelenburg, M. Theunissen, E. J. M. de Nijs, M. H. J. van den Beuken-van Everdingen and Y. M. van der Linden in Palliative Medicine

sj-docx-2-pmj-10.1177_02692163261434188 – Supplemental material for Dry mouth in palliative care: A systematic review of clinical practice guidelines around the world Supplemental material, sj-docx-2-pmj-10.1177_02692163261434188 for Dry mouth in palliative care: A systematic review of clinical practice guidelines around the world by A. I. van der Meulen, A. Stoppelenburg, M. Theunissen, E. J. M. de Nijs, M. H. J. van den Beuken-van Everdingen and Y. M. van der Linden in Palliative Medicine

sj-docx-3-pmj-10.1177_02692163261434188 – Supplemental material for Dry mouth in palliative care: A systematic review of clinical practice guidelines around the world Supplemental material, sj-docx-3-pmj-10.1177_02692163261434188 for Dry mouth in palliative care: A systematic review of clinical practice guidelines around the world by A. I. van der Meulen, A. Stoppelenburg, M. Theunissen, E. J. M. de Nijs, M. H. J. van den Beuken-van Everdingen and Y. M. van der Linden in Palliative Medicine

sj-docx-4-pmj-10.1177_02692163261434188 – Supplemental material for Dry mouth in palliative care: A systematic review of clinical practice guidelines around the world Supplemental material, sj-docx-4-pmj-10.1177_02692163261434188 for Dry mouth in palliative care: A systematic review of clinical practice guidelines around the world by A. I. van der Meulen, A. Stoppelenburg, M. Theunissen, E. J. M. de Nijs, M. H. J. van den Beuken-van Everdingen and Y. M. van der Linden in Palliative Medicine
