# Supplementary material for: Dry mouth in palliative care: A systematic review of clinical practice guidelines around the world
Source: Palliat Med. 2026 Apr 29;40(7):933–57. doi: 10.1177/02692163261434188 (PMC13323937; doi:10.1177/02692163261434188)
Supplement: sj-docx-1-pmj-10.1177_02692163261434188 – Supplemental material for Dry mouth in palliative care: A systematic review of clinical practice guidelines around the world [file sj-docx-1-pmj-10.1177_02692163261434188.docx]

**Supplementary File 4
AGREE II results and rater reliability**

**Quality appraisal of clinical practice guidelines using AGREE II criteria by 2 independent appraisers (n=72)**

| **Clinical practice guideline** | **Domain scores** | | | | | | **Overall score** | **Recommendation**  for use based on quality per AGREE II criteria |
| --- | --- | --- | --- | --- | --- | --- | --- | --- |
|  | **Scope and purpose**  (items 1-3)  % | **Stakeholder involvement**  (items 4-6)  % | **Rigour of development** (items 7-14)  % | **Clarity of presentation** (items 15-17)  % | **Applicability**  (items 18-21)  % | **Editorial Independence** (item 22-23)  % | on a scale of 1-7  (1= lowest, 7=highest) |  |
| Albania^1^ | 67 | 42 | 9 | 67 | 10 | 0 | 2 | No |
| Albania^2^ | 47 | 47 | 8 | 53 | 2 | 13 | 2 | No |
| Argentina^3^ | 50 | 28 | 10 | 72 | 4 | 8 | 2 | No |
| Argentina^4^ | 53 | 47 | 10 | 75 | 2 | 0 | 2 | No |
| Australia^5^ | 67 | 28 | 18 | 83 | 6 | 0 | 3 | No |
| Australia^6^ | 78 | 36 | 28 | 78 | 75 | 13 | 3 | No |
| Australia^7^ | 75 | 33 | 7 | 69 | 0 | 0 | 3 | No |
| Belarus^8^ | 64 | 22 | 3 | 72 | 0 | 0 | 1 | No |
| Brazil^9^ | 28 | 42 | 14 | 61 | 0 | 0 | 2 | No |
| Brazil^10^ | 56 | 25 | 11 | 69 | 0 | 0 | 2 | No |
| Bulgaria^11^ | 31 | 22 | 6 | 44 | 10 | 0 | 1 | No |
| Cameroon^12^ | 56 | 42 | 2 | 67 | 0 | 0 | 2 | No |
| Canada^13^ | 64 | 3 | 0 | 81 | 15 | 0 | 2 | No |
| Canada^14^ | 100 | 50 | 59 | 83 | 4 | 50 | 5 | Yes with modifications |
| Canada^15^ | 28 | 36 | 40 | 81 | 17 | 75 | 3 | No |
| Canada^16^ | 28 | 17 | 8 | 78 | 8 | 0 | 2 | No |
| Canada^17^ | 67 | 42 | 22 | 86 | 13 | 0 | 3 | No |
| Chile^18^ | 64 | 53 | 18 | 67 | 29 | 38 | 3 | No |
| Chile^19^ | 86 | 42 | 16 | 64 | 0 | 21 | 3 | No |
| China^20^ | 67 | 47 | 31 | 78 | 0 | 4 | 3 | No |
| Columbia^21^ | 72 | 89 | 65 | 94 | 77 | 100 | 5 | Yes with modifications |
| Costa Rica^22^ | 64 | 19 | 4 | 56 | 6 | 0 | 1 | No |
| Denmark^23^ | 69 | 36 | 19 | 86 | 21 | 63 | 3 | No |
| Denmark^24^ | 78 | 42 | 26 | 86 | 15 | 17 | 4 | No |
| Ecuador^25^ | 94 | 78 | 60 | 89 | 23 | 63 | 4 | Yes with modifications |
| Estonia^26^ | 100 | 83 | 85 | 94 | 81 | 54 | 6 | Yes with modifications |
| eSwatini^27^ | 58 | 25 | 4 | 58 | 6 | 8 | 2 | No |
| Ethiopia^28^ | 61 | 25 | 3 | 64 | 6 | 0 | 2 | No |
| Europe^29^ | 89 | 56 | 26 | 89 | 2 | 13 | 3 | No |
| Finland^30^ | 94 | 69 | 44 | 94 | 23 | 54 | 4 | No |
| Finland^31^ | 81 | 47 | 74 | 94 | 38 | 42 | 5 | Yes with modifications |
| France^32^ | 31 | 11 | 4 | 92 | 8 | 13 | 2 | No |
| Germany^33^ | 100 | 92 | 99 | 100 | 67 | 92 | 6 | Yes |
| Hungary^34^ | 72 | 72 | 41 | 83 | 40 | 63 | 4 | No |
| India^35^ | 50 | 44 | 9 | 69 | 0 | 0 | 2 | No |
| International^36^ | 56 | 25 | 45 | 81 | 2 | 17 | 4 | No |
| International^37^ | 97 | 75 | 78 | 94 | 56 | 79 | 6 | Yes with modifications |
| International^38^ | 58 | 28 | 51 | 81 | 8 | 50 | 4 | No |
| International^39^ | 67 | 47 | 3 | 47 | 0 | 33 | 3 | No |
| International^40^ | 64 | 36 | 13 | 58 | 0 | 38 | 3 | No |
| Italy^41^ | 78 | 53 | 11 | 81 | 2 | 25 | 3 | No |
| Japan^42^ | 94 | 56 | 67 | 97 | 6 | 92 | 5 | Yes with modifications |
| Latin America ^43^ | 56 | 53 | 10 | 72 | 10 | 0 | 3 | No |
| Malaysia^44^ | 44 | 22 | 6 | 75 | 0 | 0 | 2 | No |
| Mexico^45^ | 92 | 50 | 69 | 89 | 15 | 54 | 5 | Yes with modifications |
| Mexico^46^ | 8 | 11 | 4 | 42 | 19 | 0 | 1 | No |
| Netherlands^47^ | 100 | 89 | 80 | 97 | 54 | 88 | 6 | Yes with modifications |
| Netherlands^48^ | 83 | 100 | 93 | 100 | 67 | 92 | 6 | Yes |
| New Zealand^49^ | 72 | 42 | 16 | 81 | 6 | 0 | 4 | No |
| New Zealand^50^ | 83 | 75 | 13 | 81 | 6 | 0 | 4 | No |
| Norway (2019)^51^ | 89 | 67 | 76 | 78 | 21 | 63 | 4 | Yes with modifications |
| Portugal^52^ | 53 | 33 | 10 | 89 | 2 | 17 | 3 | No |
| Saudi Arabia^53^ | 89 | 56 | 23 | 81 | 10 | 0 | 3 | No |
| Scotland^54^ | 89 | 75 | 19 | 97 | 21 | 4 | 4 | No |
| South Africa^55^ | 50 | 19 | 10 | 75 | 2 | 0 | 3 | No |
| Spain^56^ | 100 | 92 | 77 | 100 | 54 | 92 | 6 | Yes with modifications |
| Spain^57^ | 97 | 78 | 95 | 100 | 94 | 75 | 6 | Yes with modifications |
| Spain^58^ | 50 | 50 | 8 | 78 | 0 | 0 | 2 | No |
| Spain^59^ | 39 | 33 | 10 | 81 | 10 | 21 | 3 | No |
| Sri Lanka^60^ | 61 | 56 | 9 | 56 | 0 | 0 | 2 | No |
| Uganda^61^ | 53 | 42 | 4 | 69 | 6 | 4 | 2 | No |
| Uganda^62^ | 44 | 36 | 8 | 75 | 4 | 4 | 2 | No |
| Uganda^63^ | 31 | 0 | 0 | 69 | 0 | 0 | 1 | No |
| United Kingdom^64^ | 100 | 58 | 94 | 97 | 90 | 67 | 6 | Yes with modifications |
| United Kingdom^65^ | 72 | 42 | 13 | 92 | 19 | 0 | 4 | No |
| United Kingdom^66^ | 56 | 28 | 11 | 72 | 13 | 0 | 3 | No |
| United Kingdom^67^ | 92 | 75 | 76 | 97 | 46 | 100 | 5 | Yes with modifications |
| United Republic of Tanzania^68^ | 56 | 33 | 11 | 67 | 2 | 0 | 2 | No |
| United States of America^69^ | 19 | 6 | 15 | 64 | 0 | 0 | 2 | No |
| Uruguay^70^ | 53 | 22 | 2 | 61 | 8 | 0 | 1 | No |
| Venezuela^71^ | 42 | 42 | 13 | 75 | 4 | 4 | 3 | No |
| Vietnam^72^ | 53 | 47 | 4 | 67 | 0 | 0 | 2 | No |

**Rater reliability per AGREE II Domain (number of appraisers = 2)**

The rater reliability (consistency of agreement, two-way mixed effects ICC) was consistently high for each domain of the AGREE II (ICC = 0.79-0.96, p<0.001).

| **Domain** | **ICC (95% CI)** | **Significance level (p)** |
| --- | --- | --- |
| 1: Scope and Purpose | 0.807 (0.728-0.868) | <0.001*** |
| 2: Stakeholder Involvement | 0.787 (0.701-0.855) | <0.001*** |
| 3: Rigour of Development | 0.964 (0.951-0.975) | <0.001*** |
| 4: Clarity of Presentation | 0.822 (0.750-0.879) | <0.001*** |
| 5: Applicability | 0.916 (0.884-0.943) | <0.001*** |
| 6: Editorial Independence | 0.872 (0.816-0.914) | <0.001*** |

**References**

1. Shoqata Shqiptare e Kujdesit Paliativ. *Udhërrëfyes i Praktikës Klinike: Kujdesi Paliativ për Pacientët Adultë*. 2012.

2. Shoqata Shqiptare e Kujdesit Paliativ. *Protokollet klinike të kujdesit paliativ*. 2014.

3. Fundación FEMEBA. Cuidado paliativo: Guías de tratamiento para enfermería, (2004).

4. Ciudad Autónoma de Buenos Aires: Instituto Nacional del Cáncer. Manual de cuidados paliativos para la atención primaria de la salud, <https://www.argentina.gob.ar/sites/default/files/2019/04/manual_de_cuidados_paliativos_para_atencion_primaria_de_la_salud.pdf> (2025).

5. Calvary Community Team and GPs/Central and Eastern Sydney PHN. PALLIATIVE CARE CLINICAL GUIDELINES, <https://cesphn.org.au/wp-content/uploads/2022/08/Calvary_Clinical_Guidelines-WEB.pdf> (2017).

6. State of Queensland (Queensland Health). Care Plan for the Dying Person: Health Professional Guidelines. 2019.

7. Australian Commission on Safety and Quality in Health Care. End-of-life care: clinical basics, <https://www.safetyandquality.gov.au/sites/default/files/2020-11/End-of-life%20care%20-%20clinical%20basics.pdf> (2020).

8. Ministry of Health Republic of Belarus. Pharmacotherapy of the main pathological symptoms (syndromes) during the provision of palliative medical care to patients (adult population) in inpatient, outpatient and at home settings. 2022.

9. Academia Nacional de Cuidados Paliativos. Manual de Cuidados Paliativos ANCP, <https://biblioteca.cofen.gov.br/wp-content/uploads/2017/05/Manual-de-cuidados-paliativos-ANCP.pdf> (2012).

10. Ministério da Saúde Brasil. Manual de 2ª edição revisada e ampliada: Cuidados Paliativos, <https://www.gov.br/saude/pt-br/centrais-de-conteudo/publicacoes/guias-e-manuais/2023/manual-de-cuidados-paliativos-2a-edicao/view> (2023).

11. Ministry of Health Bulgaria. Palliative care for patients with oncology diseases, (2019).

12. Antolín C, García R, Gutiérrez A, et al. Guide de Poche de Soins Palliatifs, <https://paliativossinfronteras.org/nuevo-libro-de-paliativos-sin-fronteras-en-frances/> (2021).

13. Fraser Health. Oral Health: Adult – Integrated Standards for Residential Care Facilities and Group Homes, <https://www.fraserhealth.ca/-/media/Project/FraserHealth/FraserHealth/Health-Professionals/Student-Practice-Education/201810_clinical_protocol_oral_health_adult_integrated_standards_for_residential_care.pdf> (2017).

14. Cancer Care Alberta. Oral and Dental Care Management in Head and Neck Cancer, (2017).

15. Alberta Health Services. Oral Care Management Tips for Healthcare Professionals: Mucositis, Candidiasis, Xerostomia, <https://www.albertahealthservices.ca/assets/info/hp/cancer/if-hp-cancer-guide-symptom-summary-oral-care.pdf> (2019).

16. British Columbia Cancer. Symptom Management Guidelines: XEROSTOMIA, <http://www.bccancer.bc.ca/nursing-site/documents/18.%20xerostomia.pdf> (2019).

17. Ontario Health - Cancer Care Ontario. Symptom Management Algorithm: Xerostomia & Salivary Hypofunction In Adults with Cancer, <https://www.cancercareontario.ca/en/system/files_force/symptoms/XerostomiaAndSalivaryHypofunctionAlgorithm.pdf?download=1> (2021).

18. Ministerio de Salud Chile. Orientación técnica cuidados paliativos universales, (2022).

19. Pontificia Universidad Católica de Chile. Evaluación y manejo de pacientes en fin de vida. 2023.

20. General Practice Branch of Cross-Strait Medicine Exchange Association. Chinese Guideline for Use of Essential Medicines in Palliative and Hospice Care. *Chinese General Practice* 2021; 24: 1717–1734. DOI: 10.12114/j.issn.1007-9572.2021.00.418.

21. Sistema General de Seguridad Social en Salud Colombia. Guía de Práctica Clínica para la atención de pacientes en Cuidado Paliativo (adopción), <https://www.minsalud.gov.co/sites/rid/Lists/BibliotecaDigital/RIDE/DE/CA/gpc-completa-cuidados-paliativos-adopcion.pdf> (2016).

22. Ministerio de Salud Costa Rica. Manual De Normas De Atención Del Dolor Y Cuidados Paliativos Del I Y Ii Nivel, <https://www.medicos.cr/website/documentos/NormativaLegal/NormativaGeneralEjercicioProfesion/Manual%20de%20Normas%20de%20Atencio%CC%81n%20del%20Dolor%20y%20Cuidados%20Paliativos%20del%20Nivel%20I%20%20y%20II.pdf> (2015).

23. Indenrigs- og Sundhedsministeriet. Mundtørhed ved fremskreden sygdom, <https://www.sundhed.dk/sundhedsfaglig/laegehaandbogen/kraeft/tilstande-og-sygdomme/palliativ-medicin/mundtoerhed-ved-fremskreden-sygdom/> (2024).

24. Dansk Selskab for Almen Medicin (DSAM). Palliativ pleje, <https://www.dsam.dk/vejledninger/palliation/symptombehandling-behandling-af-symptomer-og-tilstande#mundtoerhed> (2024).

25. Ministerio de Salud Pública del Ecuador. Cuidados paliativos. Guía de Práctica Clínica (Adopción de GPC sobre cuidados paliativos en el SNS Ministerio de Salud y Consumo, Gobierno

Español), (2014).

26. Tartu Ülikool and Tervisekassa. Palliatiivne ravi ii osa erakorraliste seisundite käsitlus, elulõpuravi ja palliatiivse ravi korraldus, <https://ravijuhend.ee/tervishoiuvarav/juhendid/150/palliatiivne-ravi-ii-osa-erakorraliste-seisundite-kasitlus-elulopuravi-ja-palliatiivse-ravi-korraldus> (2021).

27. Ministry of Health Kingdom of eSwatini. NATIONAL PALLIATIVE CARE GUIDELINES, (2011).

28. Federal Ministry of Health Ethiopia. National palliative care guideline, (2016).

29. Kossioni AE, Hajto-Bryk J, Janssens B, et al. Practical guidelines for physicians in promoting oral health in frail older adults. *Journal of the American Medical Directors Association* 2018; 19: 1039–1046.

30. Suomalaisen Lääkäriseuran Duodecimin ja Suomen Palliatiivisen Lääketieteen yhdistyksen asettama työryhmä. Palliatiivinen hoito ja saattohoito, <https://www.kaypahoito.fi/hoi50063> (2019).

31. Hotus. Palliatiivisessa hoidossa ja saattohoidossa olevan potilaan suunhoito, <https://hotus.fi/hoitosuositus/palliatiivisessa-hoidossa-ja-saattohoidossa-olevan-potilaan-suunhoito/> (2022, 2025).

32. Collège national des acteurs infirmiers groupe de travail : soins de la bouche. Le soin de bouche. 2022.

33. Deutsche Krebsgesellschaft e.V. (DKG)/Deutsche Gesellschaft fur Palliativmedizin. Erweiterte S3 Leitlinie Palliativmedizin für Patienten mit einer nicht heilbaren Krebserkrankung, <https://register.awmf.org/assets/guidelines/128-001OLl_S3_Palliativmedizin_2020-09_02.pdf> (2020).

34. Belügyminisztérium. *A Belügyminisztérium egészségügyi szakmai irányelve a daganatos felnőtt betegek teljes körű hospice és palliatív ellátásáról*. 2023.

35. Palcare and Jimmy S Bilimoria Foundation. Palliative Care Guidelines For A Home Setting In India: Oral Care, <https://guidelines.palcareindia.com/gastro-intestinal/oral-care/> (2021).

36. Davies A, Bagg J, Laverty D, et al. Salivary gland dysfunction ('dry mouth') in patients with cancer: a consensus statement. *Eur J Cancer Care (Engl)* 2010; 19: 172–177. 20090831. DOI: 10.1111/j.1365-2354.2009.01081.x.

37. Mercadante V, Jensen SB, Smith DK, et al. Salivary Gland Hypofunction and/or Xerostomia Induced by Nonsurgical Cancer Therapies: ISOO/MASCC/ASCO Guideline. *J Clin Oncol* 2021; 39: 2825–2843. 20210720. DOI: 10.1200/jco.21.01208.

38. Jones JA, Chavarri-Guerra Y, Corrêa LBC, et al. MASCC/ISOO expert opinion on the management of oral problems in patients with advanced cancer. *Support Care Cancer* 2022; 30: 8761–8773. 20220618. DOI: 10.1007/s00520-022-07211-2.

39. Hong C, Epstein JB, Jensen SB, et al. MASCC/ISOO Clinical Practice Statement: Clinical assessment of salivary gland hypofunction and xerostomia in cancer patients. *Support Care Cancer* 2024; 32: 551. 20240725. DOI: 10.1007/s00520-024-08691-0.

40. Hong C, Jensen SB, Vissink A, et al. MASCC/ISOO Clinical Practice Statement: Management of salivary gland hypofunction and xerostomia in cancer patients. *Support Care Cancer* 2024; 32: 548. 20240725. DOI: 10.1007/s00520-024-08688-9.

41. Azienda Unità Locale Socio-Sanitaria. APPROCCIO PRE-RETE E CURE PALLIATIVE DI BASE MANUALE CLINICO, <https://www.sicp.it/aggiornamento/linee-guida-bp-procedures/2024/07/approccio-pre-rete-e-cure-palliative-di-base/> (2024).

42. Japanese Society for Palliative Medicine. Clinical Guidelines for Infusion Therapy in Advanced Cancer Patients, (2013).

43. Organización Panamericana de la Salud. Cuidados paliativos: Guías para el manejo clínico, <https://www.paho.org/es/documentos/cuidados-paliativos-guias-para-manejo-clinico> (2004).

44. Katiman D, Lim R, Teoh C, et al. *Handbook of Palliative Medicine in Malaysia*. Malaysian Hospice Council, 2015.

45. Instituto Mexicano Del Seguro Social. Cuidados paliativos en pacientes adultos, (2017).

46. Consejo de Salubridad General Mexico. Guía de manejo integral de cuidados paliativos, <https://cuidadospaliativos.trabajosocial.mx/?portfolio=guia-de-manejo-integral-de-cuidados-paliativos> (2018).

47. Pallialine. Zorg in de Stervensfase, <https://palliaweb.nl/richtlijnen-palliatieve-zorg/richtlijn/stervensfase> (2023).

48. Pallialine. Multidisciplinaire richtlijn: ‘Mondproblemen in de palliatieve fase’, <https://palliaweb.nl/richtlijnen-palliatieve-zorg/richtlijn/mondproblemen-in-de-palliatieve-fase> (2025).

49. North Haven Hospice. Primary Palliative Care Guidelines, (2020).

50. Jones W and Randall C. *The Palliative Care Handbook New Zealand: First Edition*. Warrington, NZ: Hospice New Zealand Incorporated, 2024.

51. Helsedirektoret. Palliasjon i kreftomsorgen – handlingsprogram, <https://www.helsedirektoratet.no/retningslinjer/palliasjon-i-kreftomsorgen-handlingsprogram> (2019).

52. Núcleo de Estudos de Medicina Paliativa - Sociedade Portuguesa de Medicina Interna. Guia Prático do Controlo Sintomático, <https://www.spmi.pt/guia-pratico-de-controlo-sintomatico/> (2021).

53. National Cancer Center Saudia Arabia. Saudi Palliative Care National Clinical Guidelines for Oncology, <https://shc.gov.sa/Arabic/NewNCC/Documents/Palliative%20Care%20Guidelines%202019.pdf> (2019).

54. Health Improvement Scotland. Scottish Palliative Care Guidelines: Mouth Care, <https://rightdecisions.scot.nhs.uk/scottish-palliative-care-guidelines/last-days-of-life/mouth-care/> (2024).

55. Hospice Palliative Care Association of South Africa. Clinical Guidelines, (2012).

56. Grupo de Trabajo de la Guía de Práctica Clínica sobre Cuidados Paliativos. *Guía de Práctica Clínica sobre Cuidados Paliativos*. Madrid: Plan Nacional para el SNS del MSC. Agencia de Evaluación de Tecnologías Sanitarias del País Vasco, 2008.

57. Ministerio de Sanidad Santiago de Compostela Agencia de Conocimiento en Salud (ACIS). Guía de Práctica Clínica sobre atención paliativa al adulto en situación de últimos días, (2021).

58. Castillo Polo A CPB, Fernández Valverde R, Martín Hurtado A, Montoro Robles MI, Pérez Medina M. *Cuidados paliativos. Guía para Atención Primaria*. Madrid: Instituto Nacional de Gestión Sanitaria, Ministerio de Sanidad, 2021.

59. Benítez-Rosario MA ABA, González Guillermo T. *Protocolos de tratamiento en cuidados paliativos*. Barcelona: Medical Dosplus, S.L., 2023.

60. Sri Lanka Medical Association - Palliative and End-of-Life Care Task Force. Palliative Care Manual for Healthcare Professionals in Sri Lanka, <https://nccp.health.gov.lk/en/posts/palliative-care-manual-for-health-care-professionals-in-sri-lanka-2nd-edition> (2021).

61. Hospice Africa Uganda - Institute for Hospice and Palliative Care in Africa. Palliative Medicine: Pain and symptom control in the cancer and/or AIDS patient in Uganda and other African countries, (2012).

62. Ministry of Health - The Republic of Uganda. Uganda Clinical Guidelines 2023: National Guidelines for Management of Common Health Conditions, <https://library.health.go.ug/uganda-clinical-guidelines-2023> (2023).

63. Makerere Palliative Care Unit. Palliative care guidelines, (Unknown).

64. National Institute for Health and Care Excellence (NICE). Care of dying adults in the last days of life, <https://www.nice.org.uk/guidance/ng31> (2015).

65. The Royal College of Surgeons of England and The British Society for Disability and Oral Health. The Oral Management of Oncology Patients Requiring Radiotherapy, Chemotherapy and / or Bone Marrow Transplantation, <https://www.rcseng.ac.uk/-/media/files/rcs/fds/publications/rcs-oncology-guideline-update--v36.pdf> (2018).

66. UK Oral Management in Cancer Care Group (UKOMiC). Oral Care guidance and support in cancer and palliative care, <http://ukomic.com/documents/UKOMiC-Guidance-3rd-Edition.pdf> (2019).

67. National Institute for Health and Care Excellence (NICE). Palliative care - oral, cks.nice.org.uk/topics/pallitiave-care-oral (2023).

68. The United Republic of Tanzania - Ministry of Health CD, Gender, Elderly and Children,. National Cancer Treatment Guidelines, (2020).

69. American Academy of Oral Medicine (AAOM). Clinical management of cancer therapy-induced salivary gland hypofunction and xerostomia, <https://www.aaom.com/assets/docs/Practice-Statements/cps%20clinical%20%20mgmt%20cancer%20therapy%202016.pdf> (2016).

70. Ministerio de Salud Uruguay. Cuidados Paliativos Control de Síntomas, (2017).

71. Sociedad Venezolana de Medicina Paliativa (SVMP). *Manual de Cuidados Paliativos para el Primer Nivel de Atención*. 2012.

72. Bộ Y tế. Quyết định số 183/QĐ-BYT năm 2022 về việc ban hành Hướng dẫn chăm sóc giảm nhẹ. Hà Nội: Bộ Y tế, 2022.
