# Supplementary material for: Dry mouth in palliative care: A systematic review of clinical practice guidelines around the world
Source: Palliat Med. 2026 Apr 29;40(7):933–57. doi: 10.1177/02692163261434188 (PMC13323937; doi:10.1177/02692163261434188)
Supplement: sj-docx-2-pmj-10.1177_02692163261434188 – Supplemental material for Dry mouth in palliative care: A systematic review of clinical practice guidelines around the world [file sj-docx-2-pmj-10.1177_02692163261434188.docx]

**Supplementary File 2
Identification of clinical practice guidelines via targeted searches & stakeholder outreach**

- Targeted (Google) searches were performed for all 183 listed countries and territories below. Searches included variations of *palliative care [country], palliative care guideline [country], xerostomia [country], dry mouth [country]* in both English and language of [country].
- An attempt to contact at least one stakeholder per country was made, with stakeholders being sourced from the IAHPC directory ^38^, the Palliative Care Atlases ^39-42^ or a key scientific publication (n=283 stakeholders). In case of no reply or inactive contact details, a second e-mail was sent or a different stakeholder was approached if available.
- Both the targeted searches and stakeholder outreach were executed between February 2024 and August 2024.

| **Country** (/other territories) | **CPGs included** through stakeholder contact OR targeted search | **Number of stakeholders contacted** | **Response** | | | **No response** | **Comments** |
| --- | --- | --- | --- | --- | --- | --- | --- |
|  |  |  | **CPGs identified/ confirmed** | **No CPGs** with dry mouth recommendations | **Unclear response** |  |  |
| Afghanistan | 0 | 0 |  |  |  |  | Contact details unavailable. |
| Albania | 2 | 1 | x |  |  |  |  |
| Algeria | 0 | 0 |  |  |  |  | Contact details unavailable. |
| Andorra | 0 | 0 |  |  |  |  | Contact details unavailable. |
| Angola | 0 | 0 |  |  |  |  | Contact details unavailable. |
| Antigua and Barbuda | 0 | 1 |  |  |  | x |  |
| Argentina | 2 | 2 | x |  |  |  |  |
| Armenia | 0 | 2 |  | x |  |  |  |
| Aruba | 0 | 1 |  |  |  | x |  |
| Australia | 3 | 4 | x (1) |  |  | x (3) |  |
| Austria | 0 | 1 |  | x |  |  | Confirmed use of German guideline. |
| Bahamas | 0 | 1 |  |  |  | x |  |
| Bangladesh | 0 | 2 |  |  |  | x |  |
| Barbados | 0 | 1 |  |  |  | x |  |
| Belarus | 1 | 1 |  |  |  |  | Contact details inactive. |
| Belgium | 0 | 3 |  | x |  |  |  |
| Belize | 0 | 1 |  | x |  |  |  |
| Benin | 0 | 1 |  |  |  | x |  |
| Bermuda | 0 | 1 |  |  |  | x |  |
| Bhutan | 0 | 1 |  |  |  | x |  |
| Bolivia | 0 | 1 |  |  |  | x |  |
| Bosnia and Herzegovina | 0 | 1 |  |  |  | x |  |
| Botswana | 0 | 2 |  |  |  | x |  |
| Brazil | 2 | 2 |  |  |  | x |  |
| Brunei | 0 | 0 |  |  |  |  | Contact details unavailable. |
| Bulgaria | 1 | 1 |  |  |  | x |  |
| Burkina Faso | 0 | 1 |  |  |  | x |  |
| Burundi | 0 | 1 |  |  |  |  | Contact details inactive. |
| Cabo Verde | 0 | 0 |  |  |  |  | Contact details unavailable. |
| Cambodia | 0 | 1 |  |  |  |  | Contact details inactive. |
| Cameroon | 1 | 3 | x |  |  | x |  |
| Canada | 5 | 5 | x |  |  |  |  |
| Cayman Islands | 0 | 1 |  |  |  | x |  |
| Central African Republic | 0 | 0 |  |  |  |  | Contact details unavailable. |
| Chad | 0 | 0 |  |  |  |  | Contact details unavailable. |
| Chile | 2 | 2 |  |  |  | x |  |
| China | 1 | 2 |  |  |  | x |  |
| Colombia | 1 | 2 |  |  |  | x |  |
| Congo | 0 | 1 |  |  |  | x |  |
| Costa Rica | 1 | 3 |  |  | x |  |  |
| Côte d’Ivoire | 0 | 1 |  |  |  | x |  |
| Croatia | 0 | 1 |  |  |  | x |  |
| Cuba | 0 | 1 |  |  |  | x |  |
| Curacao | 0 | 1 |  | x |  |  |  |
| Cyprus | 0 | 1 |  | x |  |  |  |
| Czechia | 0 | 1 |  | x |  |  |  |
| Democratic People’s Republic of Korea | 0 | 0 |  |  |  |  | Contact details unavailable. |
| Democratic Republic of the Congo | 0 | 1 |  |  |  | x |  |
| Denmark | 2 | 2 | x |  |  |  |  |
| Djibouti | 0 | 0 |  |  |  |  | Contact details unavailable. |
| Dominican Republic | 0 | 1 |  |  |  | x |  |
| Ecuador | 1 | 1 |  |  |  | x |  |
| Egypt | 0 | 1 |  | x |  |  |  |
| El Salvador | 0 | 1 |  |  |  | x |  |
| Equatorial Guinea | 0 | 0 |  |  |  |  | Contact details unavailable. |
| Eritrea | 0 | 0 |  |  |  |  | Contact details unavailable. |
| Estonia | 1 | 1 |  |  |  | x |  |
| Eswatini | 1 | 1 |  |  |  | x |  |
| Ethiopia | 1 | 1 | x |  |  |  |  |
| Fiji | 0 | 1 |  | x |  |  |  |
| Finland | 2 | 2 |  |  | x |  | Contact details inactive (1). |
| France | 1 | 1 | x |  |  |  |  |
| Gabon | 0 | 0 |  |  |  |  | Contact details unavailable. |
| Gambia (The Republic of The) | 0 | 2 |  |  |  |  | Contact details inactive. |
| Georgia | 0 | 1 |  | x |  |  |  |
| Germany | 1 | 3 | x |  |  |  |  |
| Ghana | 0 | 2 |  |  |  | x (1) | Contact details inactive (1). |
| Greece | 0 | 1 |  | x |  |  |  |
| Guam | 0 | 0 |  |  |  |  | Contact details unavailable. |
| Guatemala | 0 | 2 |  |  |  | x |  |
| Guinea | 0 | 1 |  |  |  | x |  |
| Guinea Bissau | 0 | 0 |  |  |  |  | Contact details unavailable. |
| Guyana | 0 | 1 |  |  |  | x |  |
| Haiti | 0 | 0 |  |  |  |  | Contact details unavailable. |
| Honduras | 0 | 1 |  |  |  | x |  |
| Hong Kong | 0 | 1 |  | x |  |  |  |
| Hungary | 1 | 1 | x |  |  |  |  |
| Iceland | 0 | 2 |  |  |  | x (1) | Contact details inactive (1). |
| India | 1 | 2 |  |  |  | x |  |
| Indonesia | 0 | 1 |  | x |  |  |  |
| Iran (Islamic Republic of) | 0 | 2 |  |  |  | x |  |
| Iraq |  |  |  |  |  |  |  |
| Ireland | 0 | 2 | x |  |  |  | Confirmed use of guidelines of United Kingdom Of Great Britain and Northern Ireland; and of Scotland. |
| Israel | 0 | 1 |  |  |  | x |  |
| Italy | 1 | 3 |  |  |  | x |  |
| Jamaica | 0 | 1 |  |  |  | x |  |
| Japan | 1 | 1 | x |  |  |  |  |
| Jordan | 0 | 1 |  |  |  |  | Contact details inactive. |
| Kazakhstan | 0 | 1 |  | x |  |  |  |
| Kenya | 0 | 2 |  |  |  | x |  |
| Kuwait | 0 | 0 |  |  |  |  | Contact details inactive |
| Kyrgyzstan | 0 | 1 |  |  |  | x |  |
| Lao People’s Democratic Republic | 0 | 0 |  |  |  |  | Contact details unavailable. |
| Latvia | 0 | 0 |  |  |  |  | Contact details inactive. |
| Lebanon | 0 | 2 |  | x |  |  |  |
| Lesotho | 0 | 2 |  | x |  |  |  |
| Liberia | 0 | 0 |  |  |  |  | Contact details unavailable. |
| Libya | 0 | 0 |  |  |  |  | Contact details unavailable. |
| Liechtenstein | 0 | 0 |  |  |  |  | Contact details unavailable. |
| Lithuania | 0 | 1 |  |  |  | x |  |
| Luxembourg | 0 | 1 |  |  |  | x |  |
| Madagascar | 0 | 1 |  |  |  |  | Contact details unavailable. |
| Malawi | 0 | 2 |  | x |  |  |  |
| Malaysia | 1 | 1 | x |  |  |  |  |
| Maldives | 0 | 0 |  |  |  |  | Contact details unavailable. |
| Mali | 0 | 0 |  |  |  |  | Contact details unavailable. |
| Malta | 0 | 2 |  | x |  |  |  |
| Mauritania | 0 | 0 |  |  |  |  | Contact details unavailable. |
| Mauritius | 0 | 1 |  |  |  | x |  |
| Mexico | 2 | 2 |  |  |  | x |  |
| Micronesia (Federated States of) | 0 | 0 |  |  |  |  | Contact details unavailable. |
| Mongolia | 0 | 2 |  | x |  |  |  |
| Morocco | 0 | 2 |  |  |  | x |  |
| Mozambique | 0 | 1 |  |  |  | x |  |
| Myanmar | 0 | 1 |  |  |  | x |  |
| Namibia | 0 | 1 |  |  |  | x |  |
| Nepal | 0 | 1 |  | x |  |  |  |
| Netherlands | 2 | 2 | 2 |  |  |  |  |
| New Zealand | 2 | 2 | 2 |  |  |  |  |
| Nicaragua | 0 | 0 |  |  |  |  | Contact details unavailable. |
| Niger | 0 | 1 |  | x |  |  |  |
| Nigeria | 0 | 2 |  |  |  | x |  |
| North Macedonia | 0 | 1 |  |  |  | x |  |
| Norway | 1 | 2 | x (1) |  |  | x (1) |  |
| Oman | 0 | 1 |  |  |  | x |  |
| Pakistan | 0 | 1 |  |  |  | x |  |
| Palestine | 0 | 1 |  | x |  |  |  |
| Panama | 0 | 1 |  | x |  |  |  |
| Paraguay | 0 | 1 |  |  |  | x |  |
| Peru | 0 | 1 |  |  |  | x |  |
| Philippines | 0 | 2 |  |  | x |  |  |
| Poland | 0 | 2 |  |  |  | x (1) | Contact details inactive (1). |
| Portugal | 1 | 2 |  |  |  | x |  |
| Puerto Rico | 0 | 1 |  |  |  | x |  |
| Qatar | 0 | 1 |  |  |  | x |  |
| Republic of Korea | 0 | 1 |  |  |  | x |  |
| Republic of Moldova | 0 | 1 |  |  |  |  | Contact details inactive. |
| Romania | 0 | 2 |  |  |  | x (1) | Contact details inactive (1). |
| Russian Federation | 0 | 1 |  |  |  | x |  |
| Rwanda | 0 | 2 |  | x |  |  |  |
| Saint Kitts and Nevis | 0 | 0 |  |  |  |  | Contact details unavailable. |
| Saint Lucia | 0 | 1 |  |  |  | x |  |
| Saint Vincent and the Grenadines | 0 | 0 |  |  |  |  | Contact details unavailable. |
| Samoa | 0 | 1 |  |  |  |  | Contact details inactive. |
| San Marino | 0 | 0 |  |  |  |  | Contact details unavailable. |
| Sao Tome & Principe | 0 | 0 |  |  |  |  | Contact details unavailable. |
| Saudi Arabia | 1 | 1 |  |  |  | x |  |
| Scotland | 1 | 1 | 1 |  |  |  |  |
| Senegal | 0 | 1 |  |  |  | x |  |
| Serbia | 0 | 1 |  |  |  |  | Contact details unavailable. |
| Sierra Leone | 0 | 1 |  |  |  |  | Contact details inactive. |
| Singapore | 0 | 3 |  | x (1) |  | x (2) |  |
| Sint Maarten | 0 | 1 |  | x |  |  |  |
| Slovakia | 0 | 1 |  |  |  | x |  |
| Slovenia | 0 | 1 |  | 1 |  |  |  |
| South Africa | 1 | 2 | x (1) |  |  | x (1) |  |
| South Sudan | 0 | 1 |  |  |  |  | Contact details inactive. |
| Spain | 4 | 3 | x (1) |  |  | x (2) |  |
| Sri Lanka | 1 | 1 |  |  |  | x |  |
| Sudan | 0 | 1 |  |  |  | x |  |
| Suriname | 0 | 1 |  |  |  |  | Contact details inactive. |
| Sweden | 0 | 3 |  |  |  | x |  |
| Switzerland | 0 | 2 |  | x (1) |  | x (1) |  |
| Syrian Arab Republic | 0 | 0 |  |  |  |  | Contact details unavailable. |
| Taiwan | 0 | 1 |  |  |  | x |  |
| Tajikistan | 0 | 1 |  |  |  |  | Contact details inactive. |
| Thailand | 0 | 1 |  |  |  | x |  |
| Tonga | 0 | 0 |  |  |  |  | Contact details unavailable. |
| Trinidad and Tobago | 0 | 1 |  | x |  |  |  |
| Tunisia | 0 | 1 |  |  |  | x |  |
| Türkiye | 0 | 2 |  | x |  |  |  |
| Uganda | 3 | 4 |  |  |  |  |  |
| Ukraine | 0 | 2 |  |  |  | x |  |
| United Arab Emirates | 0 | 1 |  |  |  | x |  |
| United Kingdom of Great Britain and Northern Ireland | 4 | 3 | x |  |  |  |  |
| United Republic of Tanzania | 1 | 2 |  |  |  | x |  |
| United States of America | 1 | 3 |  |  | x (1) | x (2) |  |
| Uruguay | 1 | 1 | x |  |  |  |  |
| Uzbekistan | 0 | 0 |  |  |  |  | Contact details unavailable. |
| Venezuela (Bolivian Republic of) | 1 | 1 |  |  |  | x |  |
| Vietnam | 1 | 1 | x |  |  |  |  |
| Zambia | 0 | 3 |  | x (1) |  | x (2) |  |
| Zimbabwe | 0 | 1 |  |  |  | x |  |
