# Supplementary material for: Dry mouth in palliative care: A systematic review of clinical practice guidelines around the world
Source: Palliat Med. 2026 Apr 29;40(7):933–57. doi: 10.1177/02692163261434188 (PMC13323937; doi:10.1177/02692163261434188)
Supplement: sj-docx-3-pmj-10.1177_02692163261434188 – Supplemental material for Dry mouth in palliative care: A systematic review of clinical practice guidelines around the world [file sj-docx-3-pmj-10.1177_02692163261434188.docx]

**Supplementary File 1**
**Identification of clinical practice guidelines via electronic bibliographic databases**

**Search strategy for electronic scientific databases**The search strategy for electronic scientific databases combined two search strings related to: 1) dry mouth symptoms and 2) clinical practice guidelines. Search terms related to 3) oral care were considered but not included, as pilot searches yielded a high number of irrelevant records, substantially reducing search specificity.

**PubMed**

*Accessed on 09-11-2023; updated on 28-08-25
Results (28-08-25): 306*

("Xerostomia"[Majr] OR "Xerostomia*"[TI] OR "Decreased salivary flow"[TI] OR "dry mouth"[TI] OR "hyposaliv*"[TI] OR "hypo-saliv*"[TI] OR "mouth dryness"[TI] OR "Asialia*"[TI] OR "saliva"[TI] OR "salivary"[TI] OR "dry oral cavity"[TI] OR "salivary gland hypofunction"[TI])

AND

("guideline"[pt] OR "practice guideline" [pt] OR "health planning guidelines" [Majr] OR "Consensus Development Conference"[Publication Type] OR "Consensus Development Conferences, NIH as Topic"[Majr] OR "Consensus Development Conferences as Topic"[Majr] OR "Consensus Development Conference, NIH"[Publication Type] OR "Guidelines as Topic"[Majr] OR "Practice Guidelines as Topic"[Majr] OR "Practice Guideline" [Publication Type] OR "Consensus"[ti] OR "Protocol*"[ti] OR "Standard procedure*"[ti] OR "Guid*"[ti] OR "Instruct*"[ti] OR "Policy"[ti] OR "Policies"[ti] OR "guiding Principle*"[tiab] OR "Recommendation*"[ti] OR (consensus [ti] AND statement [ti]))

NOT

( "Sjögren"[TI] OR "Sjogren"[TI])

Limit: 01-01-2000 – 28-08-2025

**Embase (through OVID)**

*Accessed on 09-11-2023; updated on 28-08-25
Results (28-08-25): 405*

| # | Searches |
| --- | --- |
| 1 | *"Xerostomia"/ or "Xerostomia*".ti. or "Decreased salivary flow".ti. or "dry mouth".ti. or "hyposaliv".ti. or "hypo-saliv*".ti. or "mouth dryness".ti. or "Asialia*".ti. or "saliva".ti. or "salivary".ti. or "dry oral cavity".ti. or "salivary gland hypofunction".ti. |
| 2 | *"Practice Guideline"/ or *”consensus”/ or *"Consensus Development"/ or ("guideline*" or "practice guideline*").ti. or "health planning guideline*".ti. or "Consensus".ti. or Protocol*.ti. or "Standard procedure*".ti. or "Guid*".ti. or "Instruct*".ti. or "Policy".ti. or "Policies".ti. or "guiding Principle*".ti. or "Recommendation*".ti. or (consensus and statement).ti. |
| 3 | ("Sjögren" or "Sjogren").ti. |
| 4 | 1 and 2 |
| 5 | 4 not 3 |
| 6 | limit 5 to (dd=20000101-20250828 or rd=20000101-20250828) |

**CINAHL**

*Accessed on: 09-11-2023; updated on 28-08-25
Results (28-08-25): 216*

((MH xerostomia) OR (TI xerostomia*) OR (AB xerostomia*) OR (TI “decreased salivary flow”) OR (TI "dry mouth") OR AB (“dry mouth”) OR (TI hyposaliv*) OR AB (hyposaliv*) OR (TI "hypo-saliv*") OR AB (hypo-saliv*) OR (TI "mouth dryness") OR AB (“mouth dryness”) OR (TI "Asialia*") OR (TI "saliva") OR (TI "salivary") OR (TI "dry oral cavity") OR (TI "salivary gland hypofunction")) )

AND

AND ( (PT protocol) OR (PT practice guidelines) OR (PT practice acts) OR (PT nurse practice acts) OR (MH “Practice Guidelines”) OR (MH "Evidence-based practice guidelines") OR (MH Consensus) OR (TI guid*) OR (TI Consensus) OR (TI Protocol*) OR (TI “Standard Procedure*”) OR (TI Instruct*) OR (TI Policy) OR (TI Policies) OR (TI “guiding principle*”) OR (TI Recommendation*) OR (TI consensus AND TI statement))

NOT

(TI Sjögren* OR TI Sjogren*)

Limit: 01-01-2000 – 28-08-2025

**Cochrane***Accessed on: 09-11-2023; updated on 28-08-25
Results (28-08-25): 822*

| 1 | (Xerostomia OR "Decreased salivary flow" OR "dry mouth" OR hyposalivation OR hyposalivating OR "hypo-salivation" OR "hypo-salivating" OR "mouth dryness" OR Asialia OR saliva OR salivary OR "dry oral cavity" OR "salivary gland hypofunction")  *in Cochrane Reviews, Cochrane Protocols, Clinical Answers* |
| --- | --- |
| 2 | ("Consensus Development" OR Consensus OR Protocol OR Protocols OR "Standard procedure" OR "Standard procedures" OR Guideline OR Guidelines OR Instruction OR Instructions OR Policy OR Policies OR "guiding Principle" OR "guiding Principles" OR Recommendation OR Recommendations OR guideline OR guidelines OR "practice guideline" OR "practice guidelines" OR (consensus AND statement ))  *in Cochrane Reviews, Cochrane Protocols, Clinical Answers* |
| 3 | (Sjögren OR Sjogren) *in Cochrane Reviews, Cochrane Protocols, Clinical Answers* |
| 4 | #1 AND #2 NOT #3 |

Limit: 01-01-2000 – 28-08-2025

**Search strategy for guideline databases**

The search strategy for guideline databases combined three search strings related to: 1) dry mouth symptoms, 2) clinical practice guidelines and 3) oral care.

**TRIP Pro***Accessed on: 07-12-2023; updated on 28-08-25
Results (28-08-25):* 395

((xerostomia OR "dry mouth" OR "oral problem")) OR (title:xerostomia OR (title:decreased AND title:salivary AND title:flow) OR (title:dry AND title:mouth) OR title:hyposalivation OR title:hyposalivating OR (title:mouth AND title:dryness) OR title:asialia OR title:saliva OR title:salivary OR (title:dry AND title:oral AND title:cavity) OR (title:salivary AND title:gland AND title:hypofunction) OR (title:oral AND title:care))

from_date:2000 to_date:2025

Filter: Guidelines

**BIGG International Database of GRADE guidelines**

*Accessed on: 07-12-2023; updated on 28-08-25
Results (28-08-25*): 17

xerostomia OR "decreased salivary flow" OR "dry mouth" OR hyposaliv* OR "mouth dryness" OR asialia OR saliva OR salivary OR "dry oral cavity" OR "salivary gland hypofunction" OR "oral problem*" OR "oral care" OR "oral health"

**ECRI**

*Accessed on: 07-12-2023; updated on 28-08-25
Results (28-08-25*): 21

xerostomia OR "decreased salivary flow" OR "dry mouth" OR hyposaliv* OR "mouth dryness" OR asialia OR saliva OR salivary OR "dry oral cavity" OR "salivary gland hypofunction" OR "oral problem*" OR "oral care" OR "oral health"

**Manual searches in guideline databases**

All databases that did not have a search string functionality have been searched with the following individual search terms:
*Xerostomia, decreased salivary flow, dry mouth, hyposalivation, hyposalivating, Mouth dryness, Asialia, Saliva, Salivary, Dry oral cavity, Salivary gland hypofunction, Oral problem, Oral care*.
In case of few results, the terms *Oral health, Mouth problem, Mouth disease* were also searched.

*Accessed on: 07-12-2023; updated on 28-08-25*

| Databases | Results (28-08-25) |
| --- | --- |
| Arbeitsgemeinschaft der Wissenschaftlichen Medizinischen Fachgesellschaften (AWMF) Leitlinien Register | 107 |
| GIN International Guidelines Library | 46 |
| GRADEpro GDT database | 0 |
| Scottish Intercollegiate Guidelines Network (SIGN)* | 48 |
| U.S. Preventive Services Task Force (USPSTF) Database* | 82 |
| WHO Guidelines | 0 |
| Total | 283 |
| *Search function did not work optimally, all active guidelines (with adult patient population) in the database were screened instead. | |
