# Supplementary material for: Dry mouth in palliative care: A systematic review of clinical practice guidelines around the world
Source: Palliat Med. 2026 Apr 29;40(7):933–57. doi: 10.1177/02692163261434188 (PMC13323937; doi:10.1177/02692163261434188)
Supplement: sj-docx-4-pmj-10.1177_02692163261434188 – Supplemental material for Dry mouth in palliative care: A systematic review of clinical practice guidelines around the world [file sj-docx-4-pmj-10.1177_02692163261434188.docx]

**Supplementary File 3**

**Guideline characteristics of the included clinical practice guidelines (n=72)**

| **Clinical practice guideline per country** | **Institution / Authors** | **Title of Guideline (translated)** | **Country or region** | **Year of publication** | **Language** | **Clinical practice guideline topic**  (dry mouth, oral care, oral problems, palliative care, pharmacology, others) | **Target patients**  (all life-limiting diseases, cancer, frailty) | **Comprehensiveness of dry mouth topic** (limited, moderate, extensive) | **Confirmed in use by stakeholder(s)** |
| --- | --- | --- | --- | --- | --- | --- | --- | --- | --- |
| Albania^1^ | Albanian Palliative Care Association | Clinical Practice Guideline: Palliative Care for Adult Patients | Albania | 2012 | Albanian | Palliative care | All life-limiting diseases | Limited | Confirmed |
| Albania^2^ | Albanian Palliative Care Association | Clinical protocols of Palliative Care | Albania | 2014 | Albanian | Palliative care | All life-limiting diseases | Limited | Confirmed |
| Argentina^3^ | Fundación FEMEBA | Palliative Care: Treatment Guidelines for Nursing | Argentina | 2004 | Spanish | Palliative care | All life-limiting diseases | Limited | Confirmed |
| Argentina^4^ | Ciudad Autónoma de Buenos Aires: Instituto Nacional del Cáncer | Palliative care manual for primary health care | Argentina | 2025 | Spanish | Palliative care | All life-limiting diseases | Limited | Confirmed |
| Australia^5^ | Calvary Community Team and GPs/ Central and Eastern Sydney PHN | Palliative Care Clinical Guidelines | Australia | 2017 | English | Palliative care | All life-limiting diseases | Limited | Confirmed |
| Australia^6^ | State of Queensland (Queensland Health) | Care Plan for the Dying Person: Health Professional Guidelines | Australia | 2019 | English | Palliative care (end-of-life care) | All life-limiting diseases | Limited | Confirmed |
| Australia^7^ | Australian Commission on Safety and Quality in Health Care | Comprehensive Care Standard – EoL care: clinical basics | Australia | 2020 | English | Palliative care (end-of-life care) | All life-limiting diseases | Limited | Confirmed |
| Belarus^8^ | Ministry of Health, Republic of Belarus | Clinical Protocol: Pharmacotherapy of the main pathological symptoms during the provision of palliative care to patients in inpatient, outpatient and home settings | Belarus | 2022 | Belarussian | Pharmacology | All life-limiting diseases | Limited | Unconfirmed |
| Brazil^9^ | Academia Nacional de Cuidados Paliativos / National Academy of Palliative Care | ANCP Manual of Palliative Care, 2nd edition | Brazil | 2012 | Portuguese | Palliative care | All life-limiting diseases | Moderate | Unconfirmed |
| Brazil^10^ | Ministério de Saúde | Manual of Palliative Care, 2nd edition | Brazil | 2023 | Portuguese | Palliative care | All life-limiting diseases | Limited | Unconfirmed |
| Bulgaria^11^ | Ministry of Health, Bulgaria | Palliative Care for Patients with Oncological Diseases | Bulgaria | 2019 | Bulgarian | Palliative care | (Advanced) Cancer | Limited | Unconfirmed |
| Cameroon^12^ | Antolín et al. | The Pocket Guide to Palliative Care | Cameroon | 2013 | French | Palliative care | All life-limiting diseases | Limited | Confirmed |
| Canada^13^ | Fraser Health | Oral Health: Adult – Integrated Standards for Residential Care Facilities and Group Homes | Canada | 2017 | English | Oral care | Frailty | Moderate | Confirmed |
| Canada^14^ | Cancer Care Alberta | Oral and Dental Care Management in Head and Neck Cancer | English | 2017 | English | Oral care | (Advanced) Cancer | Moderate | Confirmed |
| Canada^15^ | Alberta Health Services | Oral Care Management Tips for Healthcare Professionals: Mucositis, Candidiasis, Xerostomia | Canada | 2019 | English | Oral problems | (Advanced) Cancer | Moderate | Confirmed |
| Canada^16^ | British Columbia Cancer | Symptom Management Guidelines: Xerostomia | Canada | 2019 | English | Dry mouth | (Advanced) Cancer | Extensive | Confirmed |
| Canada^17^ | Ontario Health - Cancer Care Ontario | Xerostomia & Salivary Hypofunction In Adults with Cancer | Canada | 2021 | English | Dry mouth | (Advanced) Cancer | Extensive | Confirmed |
| Chile^18^ | Ministerio de Salud, Chile | Technical Guidance: Universal Palliative Care | Chile | 2022 | Spanish | Palliative care | All life-limiting diseases | Limited | Unconfirmed |
| Chile^19^ | Pontificia Universidad Católica de Chile | Evaluation and Management of Patients at the End of Life | Chile | 2023 | Spanish | Palliative care (end-of-life Care) | All life-limiting diseases | Limited | Unconfirmed |
| China^20^ | General Practice Branch of Cross-Strait Medicine Exchange Association | Chinese Guideline for Use of Essential Medicines in Palliative and Hospice Care | China | 2021 | English | Pharmacology | All life-limiting diseases | Limited | Unconfirmed |
| Columbia^21^ | Sistema General de Seguridad Social en Salud - Colombia | Clinical Practice Guideline for the care of patients in Palliative Care | Columbia | 2016 | Spanish | Palliative care | All life-limiting diseases | Limited | Unconfirmed |
| Costa Rica^22^ | Ministerio de Salud Costa Rica | Manual of Standards for Pain Care and Palliative Care: General Protocols for the Management of Symptoms and Complications of the Incurable and/or Terminal Patient | Costa Rica | 2015 | Spanish | Palliative care | All life-limiting diseases | Limited | Unconfirmed |
| Denmark^23^ | Indenrigs- og Sundhedsministeriet (Ministery of the Interior and Health) | Dry mouth in advanced disease | Denmark | 2024 | Danish | Dry mouth | All life-limiting diseases | Extensive | Confirmed |
| Denmark^24^ | Dansk Selskab for Almen Medicin (Danish Society for General Medicine) | Palliation: Symptom treatment - treatment of symptoms and conditions | Denmark | 2024 | Danish | Palliative care | All life-limiting diseases | Limited | Confirmed |
| Ecuador^25^ | Ministerio de Salud Pública del Ecuador | Palliative Care - Clinical Practice Guideline | Ecuador | 2014 | Spanish | Palliative care | All life-limiting diseases | Moderate | Unconfirmed |
| Estonia^26^ | Tartu Ülikool and Tervisekassa | Palliative care (Part II). Treatment of emergency conditions, organization of end-of-life care and palliative care. | Estonia | 2021 | Estonian | Palliative care | All life-limiting diseases | Limited | Unconfirmed |
| eSwatini^27^ | Ministry of Health (Kingdom of Swaziland/eSwatini) | National Palliative Care Guidelines | eSwatini | 2011 | English | Palliative care | All life-limiting diseases | Limited | Unconfirmed |
| Ethiopia^28^ | Federal Ministry of Health | National Palliative Care Guideline | Ethiopia | 2016 | English | Palliative care | All life-limiting diseases | Moderate | Confirmed |
| Europe^29^ | European College of Gerodontology/European Geriatric Medicine Society | Practical Guidelines for Physicians in Promoting Oral Health in Frail Older Adults | Europe | 2018 | English | Oral care | Frailty | Moderate | Unconfirmed |
| Finland^30^ | Suomalaisen Lääkäriseuran Duodecimin ja Suomen Palliatiivisen Lääketieteen yhdistyksen asettama työryhmä (Duodecim of the Finnish Medical Society and the Finnish Palliative Medicine Association) | Palliative care and hospice care | Finland | 2019 | Finnish | Palliative care | All life-limiting diseases | Limited | Unconfirmed |
| Finland^31^ | Hotus: Nursing Research Foundation | Palliative and Hospice Care: Oral Care | Finland | 2022 | Finnish | Palliative care | All life-limiting diseases | Moderate | Unconfirmed |
| France^32^ | Societe francaise d'accompagnement et de soins palliatifs | Care of the mouth | France | 2022 | French | Oral care | All life-limiting diseases | Moderate | Confirmed |
| Germany^33^ | Deutsche Krebsgesellschaft e.V. (DKG)/Deutsche Gesellschaft fur Palliativmedizin | Extended S3 guideline for palliative care for patients with incurable cancer | Germany | 2020 | German | Palliative care | (Advanced) Cancer; other life-limiting diseases | Moderate | Confirmed |
| Hungary^34^ | Belügyminisztérium (Ministry of the Interior) | Health professional guidelines of the Ministry of the Interior on comprehensive hospice and palliative care for adult cancer patients | Hungary | 2023 | Hungarian | Palliative care | (Advanced) Cancer; other life-limiting diseases | Limited | Confirmed |
| India^35^ | Palcare by The Jimmy S Bilimoria Foundation | Palliative Care Guidelines for a home setting in India | India | 2021 | English | Palliative care | All life-limiting diseases | Extensive | Unconfirmed |
| International^36^ | Davies et al. | Salivary gland dysfunction ('dry mouth') in patients with cancer: a consensus statement | International | 2010 | English | Dry mouth | (Advanced) Cancer | Extensive | Unconfirmed |
| International^37^ | Multinational Association of Supportive Care in Cancer/International Society of Oral Oncology/American Society of Clinical Oncology | Salivary Gland Hypofunction and/or Xerostomia Induced by Nonsurgical Cancer Therapies: ISOO/MASCC/ASCO Guideline | International | 2021 | English | Dry mouth | (Advanced) Cancer | Moderate | Confirmed |
| International^38^ | Multinational Association for Supportive Care in Cancer & International Society of Oral Oncology | MASCC/ISOO expert opinion on the management of oral problems in patients with advanced cancer | International | 2022 | English | Oral problems | (Advanced) Cancer | Moderate | Confirmed |
| International^39^ | Multinational Association of Supportive Care in Cancer (MASCC) /International Society of Oral Oncology/American Society of Clinical Oncology (ISOO) | MASCC/ISOO Clinical Practice Statement: Clinical assessment of salivary gland hypofunction and xerostomia in cancer patients | International | 2024 | English | Dry mouth | (Advanced) Cancer | Moderate | Confirmed |
| International^40^ | Multinational Association of Supportive Care in Cancer (MASCC) /International Society of Oral Oncology/American Society of Clinical Oncology (ISOO) | MASCC/ISOO Clinical Practice Statement: Management of salivary gland hypofunction and xerostomia in cancer patients | International | 2024 | English | Dry mouth | (Advanced) Cancer | Moderate | Confirmed |
| Italy^41^ | Azienda Unità Locale Socio-Sanitaria | Basic Palliative Care: Clinical Manual | Italy | 2024 | Italian | Palliative care | All life-limiting diseases | Limited | Unconfirmed |
| Japan^42^ | Japanese Society for Palliative Medicine | Guidelines for Fluid Therapy in Terminal Cancer Patients | Japan | 2013 | Japanese | Pharmacology | (Advanced) cancer; other life-limiting diseases | Moderate | Confirmed |
| Latin America ^43^ | Organización Panamericana de la Salud | Palliative Care: Guidelines for Clinical Management | Latin America | 2004 | Spanish | Palliative care | All life-limiting diseases | Extensive | Unconfirmed |
| Malaysia^44^ | Malaysian Hospice Council | Handbook of Palliative Medicine in Malaysia | Malaysia | 2015 | English | Palliative care | All life-limiting diseases | Moderate | Confirmed |
| Mexico^45^ | Instituto Mexicano Del Seguro Social | Palliative Care in Adult Patients | Mexico | 2017 | Spanish | Palliative care | All life-limiting diseases | Moderate | Unconfirmed |
| Mexico^46^ | Consejo de Salubridad General Mexico (General Health Council) | Comprehensive Management Guide: Palliative Care | Mexico | 2018 | Spanish | Palliative care | All life-limiting diseases | Limited | Unconfirmed |
| Netherlands^47^ | Pallialine/IKNL | Care in the last phase of life | Netherlands | 2023 | Dutch | Palliative care (end-of-life care) | All life-limiting diseases | Moderate | Confirmed |
| Netherlands^48^ | Pallialine/IKNL | Multidisciplinary guidelines: Mouth problems in the palliative phase | Netherlands | 2025 | Dutch | Oral care | All life-limiting diseases | Extensive | Confirmed |
| New Zealand^49^ | North Haven Hospice | Primary Palliative Care Guidelines, 3^rd^ edition | New Zealand | 2020 | English | Palliative care | All life-limiting diseases | Limited | Confirmed |
| New Zealand^50^ | Hospice New Zealand | The Palliative Care Handbook New Zealand: First Edition | New Zealand | 2024 | English | Palliative care | All life-limiting diseases | Limited | Confirmed |
| Norway (2019)^51^ | Helsedirektoret (Norwegian Directorate of Health) | Palliation in cancer care – action programme: National professional guideline | Norway | 2019 | Norwegian | Palliative care | (Advanced) Cancer; other life-limiting diseases | Extensive | Confirmed |
| Portugal^52^ | Núcleo de Estudos de Medicina Paliativa, Sociedade Portuguesa de Medicina Interna | Practice Guide for Symptom Control | Portugal | 2021 | Portuguese | Palliative care | All life-limiting diseases | Moderate | Confirmed |
| Saudi Arabia^53^ | National Cancer Center | Saudi Palliative Care National Clinical Guidelines for Oncology | Saudi Arabia | 2019 | English | Palliative care | (Advanced) cancer; also any life-limiting diseases | Extensive | Unconfirmed |
| Scotland^54^ | Healthcare Improvement Scotland | Scottish Palliative Care Guidelines: Mouth Care | Scotland | 2024 | English | Oral care | All life-limiting diseases | Extensive | Confirmed |
| South Africa^55^ | Hospice Palliative Care Association of South Africa (now: Association of Palliative Care Centres of South Africa) | HPCA Clinical Guidelines | South Africa | 2012 | English | Palliative care | All life-limiting diseases | Extensive | Confirmed |
| Spain^56^ | Ministerio de Salud y Consumo | Clinical Practice Guide on Palliative Care | Spain | 2008 | Spanish | Palliative care | All life-limiting diseases | Limited | Confirmed |
| Spain^57^ | Ministerio de Sanidad | Clinical Practice Guide on palliative care for adults in their final days | Spain | 2021 | Spanish | Palliative care (end-of-life care) | All life-limiting diseases | Limited | Confirmed |
| Spain^58^ | Ministerio de Sanidad | Palliative Care: Guidelines for Primary Care | Spain | 2021 | Spanish | Palliative care | All life-limiting diseases | Moderate | Confirmed |
| Spain^59^ | Benítez-Rosario et al. | Treatment Protocols in Palliative Care | Spanish | 2023 | Spanish | Palliative care | All life-limiting diseases | Moderate | Confirmed |
| Sri Lanka^60^ | Palliative and End-of-Life Care Task Force, Sri Lanka Medical Association | Palliative Care Manual for Healthcare Professionals in Sri Lanka, 2^nd^ edition | Sri Lanka | 2021 | English | Palliative care | All life-limiting diseases | Limited | Unconfirmed |
| Uganda^61^ | Hospice Africa Uganda & Institute for Hospice and Palliative Care in Africa | Palliative Medicine: Pain and Symptom Control in the Cancer and/or AIDS Patient in Uganda or other African Countries | Uganda | 2012 | English | Palliative care | (Advanced) cancer; other life-limiting diseases | Limited | Confirmed |
| Uganda^62^ | The Republic of Uganda Ministry of Health | Uganda Clinical Guidelines 2023 | Uganda | 2023 | English | Others (common health conditions) | All life-limiting diseases | Limited | Confirmed |
| Uganda^63^ | Makerere Palliative Care Unit | Palliative Care Guidelines: Constipation Management | Uganda | Not reported | English | Palliative care | All life-limiting diseases | Limited | Confirmed |
| United Kingdom^64^ | National Institute for Health and Care Excellence | Care of dying adults in the last days of life | United Kingdom | 2015 | English | Palliative care (end-of-life care) | All life-limiting diseases | Limited | Confirmed |
| United Kingdom^65^ | The Royal College of Surgeons of England and The British Society for Disability and Oral Health | The Oral Management of Oncology Patients Requiring Radiotherapy, Chemotherapy and/ or Bone Marrow Transplantation: Clinical Guidelines | United Kingdom | 2018 | English | Oral care | (Advanced) cancer | Moderate | Confirmed |
| United Kingdom^66^ | UK Oral Management in Cancer Care Group (UKOMIiC) | Oral Care guidance and support in cancer and palliative care, 3^rd^ edition | United Kingdom | 2019 | English | Oral care | (Advanced) Cancer | Moderate | Confirmed |
| United Kingdom^67^ | National Institute for Health and Care Excellence | Palliative care - oral | United Kingdom | 2023 | English | Oral care | All life-limiting diseases | Extensive | Confirmed |
| United Republic of Tanzania^68^ | The United Republic of Tanzania - Ministry of Health, Community Development, Gender, Elderly and Children | National Cancer Treatment Guidelines | Tanzania | 2020 | English | Others (oncology) | (Advanced) Cancer | Limited | Unconfirmed |
| United States of America^69^ | American Academy of Oral Medicine | Clinical management of cancer therapy-induced salivary gland hypofunction and xerostomia | United States of America (USA) | 2016 | English | Dry mouth | (Advanced) cancer | Moderate | Unconfirmed |
| Uruguay^70^ | Ministerio de Salud Uruguay | Palliative Care Symptom Control | Uruguay | 2017 | Spanish | Palliative care | All life-limiting diseases | Limited | Confirmed |
| Venezuela^71^ | BỘ Y TẾ (Ministry of Health, Vietnam) | Guidelines for Palliative Care | Vietnam | 2022 | English | Palliative care | All life-limiting diseases | Moderate | Unconfirmed |
| Vietnam^72^ | La Sociedad Venezolana de Medicina Paliativa // The Venezuelan Society of Palliative Medicine | Manual for Palliative Care | Venezuela | 2012 | Spanish | Palliative care | All life-limiting diseases | Limited | Confirmed |

**References**

1. Shoqata Shqiptare e Kujdesit Paliativ. *Udhërrëfyes i Praktikës Klinike: Kujdesi Paliativ për Pacientët Adultë*. 2012.

2. Shoqata Shqiptare e Kujdesit Paliativ. *Protokollet klinike të kujdesit paliativ*. 2014.

3. Fundación FEMEBA. Cuidado paliativo: Guías de tratamiento para enfermería, (2004).

4. Ciudad Autónoma de Buenos Aires: Instituto Nacional del Cáncer. Manual de cuidados paliativos para la atención primaria de la salud, <https://www.argentina.gob.ar/sites/default/files/2019/04/manual_de_cuidados_paliativos_para_atencion_primaria_de_la_salud.pdf> (2025).

5. Calvary Community Team and GPs/Central and Eastern Sydney PHN. PALLIATIVE CARE CLINICAL GUIDELINES, <https://cesphn.org.au/wp-content/uploads/2022/08/Calvary_Clinical_Guidelines-WEB.pdf> (2017).

6. State of Queensland (Queensland Health). Care Plan for the Dying Person: Health Professional Guidelines. 2019.

7. Australian Commission on Safety and Quality in Health Care. End-of-life care: clinical basics, <https://www.safetyandquality.gov.au/sites/default/files/2020-11/End-of-life%20care%20-%20clinical%20basics.pdf> (2020).

8. Ministry of Health Republic of Belarus. Pharmacotherapy of the main pathological symptoms (syndromes) during the provision of palliative medical care to patients (adult population) in inpatient, outpatient and at home settings. 2022.

9. Academia Nacional de Cuidados Paliativos. Manual de Cuidados Paliativos ANCP, <https://biblioteca.cofen.gov.br/wp-content/uploads/2017/05/Manual-de-cuidados-paliativos-ANCP.pdf> (2012).

10. Ministério da Saúde Brasil. Manual de 2ª edição revisada e ampliada: Cuidados Paliativos, <https://www.gov.br/saude/pt-br/centrais-de-conteudo/publicacoes/guias-e-manuais/2023/manual-de-cuidados-paliativos-2a-edicao/view> (2023).

11. Ministry of Health Bulgaria. Palliative care for patients with oncology diseases, (2019).

12. Antolín C, García R, Gutiérrez A, et al. Guide de Poche de Soins Palliatifs, <https://paliativossinfronteras.org/nuevo-libro-de-paliativos-sin-fronteras-en-frances/> (2021).

13. Fraser Health. Oral Health: Adult – Integrated Standards for Residential Care Facilities and Group Homes, <https://www.fraserhealth.ca/-/media/Project/FraserHealth/FraserHealth/Health-Professionals/Student-Practice-Education/201810_clinical_protocol_oral_health_adult_integrated_standards_for_residential_care.pdf> (2017).

14. Cancer Care Alberta. Oral and Dental Care Management in Head and Neck Cancer, (2017).

15. Alberta Health Services. Oral Care Management Tips for Healthcare Professionals: Mucositis, Candidiasis, Xerostomia, <https://www.albertahealthservices.ca/assets/info/hp/cancer/if-hp-cancer-guide-symptom-summary-oral-care.pdf> (2019).

16. British Columbia Cancer. Symptom Management Guidelines: XEROSTOMIA, <http://www.bccancer.bc.ca/nursing-site/documents/18.%20xerostomia.pdf> (2019).

17. Ontario Health - Cancer Care Ontario. Symptom Management Algorithm: Xerostomia & Salivary Hypofunction In Adults with Cancer, <https://www.cancercareontario.ca/en/system/files_force/symptoms/XerostomiaAndSalivaryHypofunctionAlgorithm.pdf?download=1> (2021).

18. Ministerio de Salud Chile. Orientación técnica cuidados paliativos universales, (2022).

19. Pontificia Universidad Católica de Chile. Evaluación y manejo de pacientes en fin de vida. 2023.

20. General Practice Branch of Cross-Strait Medicine Exchange Association. Chinese Guideline for Use of Essential Medicines in Palliative and Hospice Care. *Chinese General Practice* 2021; 24: 1717-1734. DOI: 10.12114/j.issn.1007-9572.2021.00.418.

21. Sistema General de Seguridad Social en Salud Colombia. Guía de Práctica Clínica para la atención de pacientes en Cuidado Paliativo (adopción), <https://www.minsalud.gov.co/sites/rid/Lists/BibliotecaDigital/RIDE/DE/CA/gpc-completa-cuidados-paliativos-adopcion.pdf> (2016).

22. Ministerio de Salud Costa Rica. Manual De Normas De Atención Del Dolor Y Cuidados Paliativos Del I Y Ii Nivel, <https://www.medicos.cr/website/documentos/NormativaLegal/NormativaGeneralEjercicioProfesion/Manual%20de%20Normas%20de%20Atencio%CC%81n%20del%20Dolor%20y%20Cuidados%20Paliativos%20del%20Nivel%20I%20%20y%20II.pdf> (2015).

23. Indenrigs- og Sundhedsministeriet. Mundtørhed ved fremskreden sygdom, <https://www.sundhed.dk/sundhedsfaglig/laegehaandbogen/kraeft/tilstande-og-sygdomme/palliativ-medicin/mundtoerhed-ved-fremskreden-sygdom/> (2024).

24. Dansk Selskab for Almen Medicin (DSAM). Palliativ pleje, <https://www.dsam.dk/vejledninger/palliation/symptombehandling-behandling-af-symptomer-og-tilstande#mundtoerhed> (2024).

25. Ministerio de Salud Pública del Ecuador. Cuidados paliativos. Guía de Práctica Clínica (Adopción de GPC sobre cuidados paliativos en el SNS Ministerio de Salud y Consumo, Gobierno

Español), (2014).

26. Tartu Ülikool and Tervisekassa. Palliatiivne ravi ii osa erakorraliste seisundite käsitlus, elulõpuravi ja palliatiivse ravi korraldus, <https://ravijuhend.ee/tervishoiuvarav/juhendid/150/palliatiivne-ravi-ii-osa-erakorraliste-seisundite-kasitlus-elulopuravi-ja-palliatiivse-ravi-korraldus> (2021).

27. Ministry of Health Kingdom of eSwatini. NATIONAL PALLIATIVE CARE GUIDELINES, (2011).

28. Federal Ministry of Health Ethiopia. National palliative care guideline, (2016).

29. Kossioni AE, Hajto-Bryk J, Janssens B, et al. Practical guidelines for physicians in promoting oral health in frail older adults. *Journal of the American Medical Directors Association* 2018; 19: 1039-1046.

30. Suomalaisen Lääkäriseuran Duodecimin ja Suomen Palliatiivisen Lääketieteen yhdistyksen asettama työryhmä. Palliatiivinen hoito ja saattohoito, <https://www.kaypahoito.fi/hoi50063> (2019).

31. Hotus. Palliatiivisessa hoidossa ja saattohoidossa olevan potilaan suunhoito, <https://hotus.fi/hoitosuositus/palliatiivisessa-hoidossa-ja-saattohoidossa-olevan-potilaan-suunhoito/> (2022, 2025).

32. Collège national des acteurs infirmiers groupe de travail : soins de la bouche. Le soin de bouche. 2022.

33. Deutsche Krebsgesellschaft e.V. (DKG)/Deutsche Gesellschaft fur Palliativmedizin. Erweiterte S3 Leitlinie Palliativmedizin für Patienten mit einer nicht heilbaren Krebserkrankung, <https://register.awmf.org/assets/guidelines/128-001OLl_S3_Palliativmedizin_2020-09_02.pdf> (2020).

34. Belügyminisztérium. *A Belügyminisztérium egészségügyi szakmai irányelve a daganatos felnőtt betegek teljes körű hospice és palliatív ellátásáról*. 2023.

35. Palcare and Jimmy S Bilimoria Foundation. Palliative Care Guidelines For A Home Setting In India: Oral Care, <https://guidelines.palcareindia.com/gastro-intestinal/oral-care/> (2021).

36. Davies A, Bagg J, Laverty D, et al. Salivary gland dysfunction ('dry mouth') in patients with cancer: a consensus statement. *Eur J Cancer Care (Engl)* 2010; 19: 172-177. 20090831. DOI: 10.1111/j.1365-2354.2009.01081.x.

37. Mercadante V, Jensen SB, Smith DK, et al. Salivary Gland Hypofunction and/or Xerostomia Induced by Nonsurgical Cancer Therapies: ISOO/MASCC/ASCO Guideline. *J Clin Oncol* 2021; 39: 2825-2843. 20210720. DOI: 10.1200/jco.21.01208.

38. Jones JA, Chavarri-Guerra Y, Corrêa LBC, et al. MASCC/ISOO expert opinion on the management of oral problems in patients with advanced cancer. *Support Care Cancer* 2022; 30: 8761-8773. 20220618. DOI: 10.1007/s00520-022-07211-2.

39. Hong C, Epstein JB, Jensen SB, et al. MASCC/ISOO Clinical Practice Statement: Clinical assessment of salivary gland hypofunction and xerostomia in cancer patients. *Support Care Cancer* 2024; 32: 551. 20240725. DOI: 10.1007/s00520-024-08691-0.

40. Hong C, Jensen SB, Vissink A, et al. MASCC/ISOO Clinical Practice Statement: Management of salivary gland hypofunction and xerostomia in cancer patients. *Support Care Cancer* 2024; 32: 548. 20240725. DOI: 10.1007/s00520-024-08688-9.

41. Azienda Unità Locale Socio-Sanitaria. APPROCCIO PRE-RETE E CURE PALLIATIVE DI BASE MANUALE CLINICO, <https://www.sicp.it/aggiornamento/linee-guida-bp-procedures/2024/07/approccio-pre-rete-e-cure-palliative-di-base/> (2024).

42. Japanese Society for Palliative Medicine. Clinical Guidelines for Infusion Therapy in Advanced Cancer Patients, (2013).

43. Organización Panamericana de la Salud. Cuidados paliativos: Guías para el manejo clínico, <https://www.paho.org/es/documentos/cuidados-paliativos-guias-para-manejo-clinico> (2004).

44. Katiman D, Lim R, Teoh C, et al. *Handbook of Palliative Medicine in Malaysia*. Malaysian Hospice Council, 2015.

45. Instituto Mexicano Del Seguro Social. Cuidados paliativos en pacientes adultos, (2017).

46. Consejo de Salubridad General Mexico. Guía de manejo integral de cuidados paliativos, <https://cuidadospaliativos.trabajosocial.mx/?portfolio=guia-de-manejo-integral-de-cuidados-paliativos> (2018).

47. Pallialine. Zorg in de Stervensfase, <https://palliaweb.nl/richtlijnen-palliatieve-zorg/richtlijn/stervensfase> (2023).

48. Pallialine. Multidisciplinaire richtlijn: ‘Mondproblemen in de palliatieve fase’, <https://palliaweb.nl/richtlijnen-palliatieve-zorg/richtlijn/mondproblemen-in-de-palliatieve-fase> (2025).

49. North Haven Hospice. Primary Palliative Care Guidelines, (2020).

50. Jones W and Randall C. *The Palliative Care Handbook New Zealand: First Edition*. Warrington, NZ: Hospice New Zealand Incorporated, 2024.

51. Helsedirektoret. Palliasjon i kreftomsorgen – handlingsprogram, <https://www.helsedirektoratet.no/retningslinjer/palliasjon-i-kreftomsorgen-handlingsprogram> (2019).

52. Núcleo de Estudos de Medicina Paliativa - Sociedade Portuguesa de Medicina Interna. Guia Prático do Controlo Sintomático, <https://www.spmi.pt/guia-pratico-de-controlo-sintomatico/> (2021).

53. National Cancer Center Saudia Arabia. Saudi Palliative Care National Clinical Guidelines for Oncology, <https://shc.gov.sa/Arabic/NewNCC/Documents/Palliative%20Care%20Guidelines%202019.pdf> (2019).

54. Health Improvement Scotland. Scottish Palliative Care Guidelines: Mouth Care, <https://rightdecisions.scot.nhs.uk/scottish-palliative-care-guidelines/last-days-of-life/mouth-care/> (2024).

55. Hospice Palliative Care Association of South Africa. Clinical Guidelines, (2012).

56. Grupo de Trabajo de la Guía de Práctica Clínica sobre Cuidados Paliativos. *Guía de Práctica Clínica sobre Cuidados Paliativos*. Madrid: Plan Nacional para el SNS del MSC. Agencia de Evaluación de Tecnologías Sanitarias del País Vasco, 2008.

57. Ministerio de Sanidad Santiago de Compostela Agencia de Conocimiento en Salud (ACIS). Guía de Práctica Clínica sobre atención paliativa al adulto en situación de últimos días, (2021).

58. Castillo Polo A CPB, Fernández Valverde R, Martín Hurtado A, Montoro Robles MI, Pérez Medina M. *Cuidados paliativos. Guía para Atención Primaria*. Madrid: Instituto Nacional de Gestión Sanitaria, Ministerio de Sanidad, 2021.

59. Benítez-Rosario MA ABA, González Guillermo T. *Protocolos de tratamiento en cuidados paliativos*. Barcelona: Medical Dosplus, S.L., 2023.

60. Sri Lanka Medical Association - Palliative and End-of-Life Care Task Force. Palliative Care Manual for Healthcare Professionals in Sri Lanka, <https://nccp.health.gov.lk/en/posts/palliative-care-manual-for-health-care-professionals-in-sri-lanka-2nd-edition> (2021).

61. Hospice Africa Uganda - Institute for Hospice and Palliative Care in Africa. Palliative Medicine: Pain and symptom control in the cancer and/or AIDS patient in Uganda and other African countries, (2012).

62. Ministry of Health - The Republic of Uganda. Uganda Clinical Guidelines 2023: National Guidelines for Management of Common Health Conditions, <https://library.health.go.ug/uganda-clinical-guidelines-2023> (2023).

63. Makerere Palliative Care Unit. Palliative care guidelines, (Unknown).

64. National Institute for Health and Care Excellence (NICE). Care of dying adults in the last days of life, <https://www.nice.org.uk/guidance/ng31> (2015).

65. The Royal College of Surgeons of England and The British Society for Disability and Oral Health. The Oral Management of Oncology Patients Requiring Radiotherapy, Chemotherapy and / or Bone Marrow Transplantation, <https://www.rcseng.ac.uk/-/media/files/rcs/fds/publications/rcs-oncology-guideline-update--v36.pdf> (2018).

66. UK Oral Management in Cancer Care Group (UKOMiC). Oral Care guidance and support in cancer and palliative care, <http://ukomic.com/documents/UKOMiC-Guidance-3rd-Edition.pdf> (2019).

67. National Institute for Health and Care Excellence (NICE). Palliative care - oral, cks.nice.org.uk/topics/pallitiave-care-oral (2023).

68. The United Republic of Tanzania - Ministry of Health CD, Gender, Elderly and Children,. National Cancer Treatment Guidelines, (2020).

69. American Academy of Oral Medicine (AAOM). Clinical management of cancer therapy-induced salivary gland hypofunction and xerostomia, <https://www.aaom.com/assets/docs/Practice-Statements/cps%20clinical%20%20mgmt%20cancer%20therapy%202016.pdf> (2016).

70. Ministerio de Salud Uruguay. Cuidados Paliativos Control de Síntomas, (2017).

71. Sociedad Venezolana de Medicina Paliativa (SVMP). *Manual de Cuidados Paliativos para el Primer Nivel de Atención*. 2012.

72. Bộ Y tế. Quyết định số 183/QĐ-BYT năm 2022 về việc ban hành Hướng dẫn chăm sóc giảm nhẹ. Hà Nội: Bộ Y tế, 2022.
